# A Comparison of *In Vitro* Nucleosome Positioning Mapped with Chicken, Frog and a Variety of Yeast Core Histones^☆^

**DOI:** 10.1016/j.jmb.2013.07.019

**Published:** 2013-11-15

**Authors:** James Allan, Ross M. Fraser, Tom Owen-Hughes, Kevin Docherty, Vijender Singh

**Affiliations:** 1 Institute of Cell Biology, Darwin Building, King's Buildings, University of Edinburgh, Edinburgh EH9 3JR, Scotland, United Kingdom; 2 Centre for Population Health Sciences, University of Edinburgh, Teviot Place, Edinburgh EH8 9AG, Scotland, United Kingdom; 3 Centre for Gene Regulation and Expression, School of Life Sciences, University of Dundee, Dundee DD1 5EH, Scotland, United Kingdom; 4 School of Medical Sciences, University of Aberdeen, Institute of Medical Sciences, Foresterhill, Aberdeen AB25 2ZD, Scotland, United Kingdom

**Keywords:** chromatin, nucleosome positioning, core histones, histone variants, Cse4

## Abstract

Using high-throughput sequencing, we have mapped sequence-directed nucleosome positioning *in vitro* on four plasmid DNAs containing DNA fragments derived from the genomes of sheep, drosophila, human and yeast. Chromatins were prepared by reconstitution using chicken, frog and yeast core histones. We also assembled yeast chromatin in which histone H3 was replaced by the centromere-specific histone variant, Cse4. The positions occupied by recombinant frog and native chicken histones were found to be very similar. In contrast, nucleosomes containing the canonical yeast octamer or, in particular, the Cse4 octamer were assembled at distinct populations of locations, a property that was more apparent on particular genomic DNA fragments. The factors that may contribute to this variation in nucleosome positioning and the implications of the behavior are discussed.

## Highlights

• Chromatins were formed on plasmids containing fragments of sheep, drosophila, human and yeast genomic DNAs with four types of histone octamer.
• Nucleosome positioning was measured for each reconstitute.
• Although similar, the binding profiles obtained with the yeast histone octamer, including one containing Cse4, were distinct from those obtained with chick or frog histones.
• The difference in nucleosome positioning was best seen on AT-rich DNA substrates.

## Graphical Abstract

**Figure:**
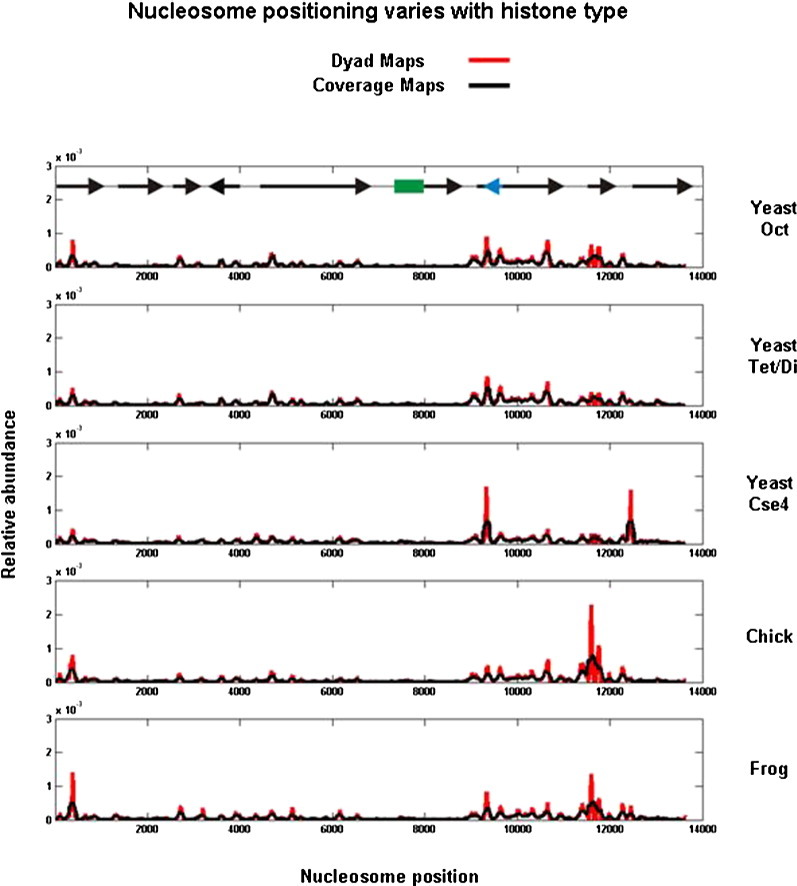

## Introduction

Recently, high-throughput analyses of genomic nucleosomal DNA sequences produced from chromatin have elucidated the role of nucleosome positioning in influencing chromatin organization, particularly in the context of gene structure and activity.

Numerous factors act to determine nucleosome positioning *in vivo*; it is widely accepted that the specificity of the binding of the core histone octamer with respect to the underlying DNA sequence plays a part [1–3]. DNA structure helps to facilitate this interaction by conferring an energetic advantage to wrapping the DNA around the histone core [4–6], by ensuring that the resulting DNA superhelix follows a left-handed path [7,8] and by accommodating irregularities in the “smoothness” of the superhelical path at sites that tend to be located at, or close to, the dyad of the nucleosome [9]. In addition, some types of sequence, poly(dA:dT), for example, resist incorporation into nucleosomes and, consequently, can have a profound influence upon nucleosome positioning [5,10]. In spite of the general acceptance of these factors, the extent to which DNA defines its own chromatin structure *in vivo* remains contentious given the influence of other factors such as chromatin remodeling, transcription factor binding and transcription itself [11–13].

In an *in vitro* system involving only histones and DNA, it has been generally assumed that DNA sequence is the sole determinant of nucleosome positioning and that the source or type of core histone octamer used has little impact upon the interaction. Thus, for example, it is seen to be acceptable to map nucleosome positioning sites on yeast genomic DNA using native chicken [3] or drosophila core histones [14] or recombinant drosophila [15] histone octamers. This is partly justified on the basis that the high-resolution structures of the core particle do not appear to vary substantially as a function of core histone source [16–20]. Consequently, the surface of the histone octamer, in terms of shape and charge distribution, is seen to present a relatively constant binding platform for the interaction with DNA, irrespective of the amino acid sequence variation exhibited by the major core histone types of eukaryotes [21]. However, it remains to be directly established whether amino acid variations between different core histone octamer types affect their DNA sequence preferences thereby influencing nucleosome positioning. Given that many other factors will modulate nucleosome positioning *in vivo* [5,22], an answer to this question is most likely to result from model *in vitro* analyses.

We have previously mapped DNA sequence-directed nucleosome positioning *in vitro* on genomic DNA sequences using high-throughput sequencing [8,23]. Our procedure involves digestion of reconstituted chromatin with a nuclease and the recovery of histone-protected nucleosomal DNA. This population of nucleosomal DNA molecules is then characterized by high-throughput sequencing that allows the histone octamer positioning sites to be mapped to the target sequence. Using this approach to map *in vitro* nucleosome positioning on relatively short DNA sequences (~ 15 kb) provides exceptionally high resolution information. Consequently, the methodology has great potential for comparative studies in which individual components or procedures can be systematically varied and their influence assessed in detail.

In the current study, we have compared DNA sequence-directed, *in vitro* nucleosome positioning as a function of the type (source) of core histone octamer used to reconstitute chromatin. We used chicken, frog and various types of yeast histones to assemble nucleosomes onto a mixture of plasmids containing human, sheep, fly and yeast genomic DNAs. The locations and relative affinities of the positioning sites for the different histone octamers were determined by high-throughput sequencing. Although all the histones tested identified similar patterns of binding sites, there was notable variation between the populations of nucleosomal DNA sequences recovered from the different reconstitutes.

## Results

### Preparation and general properties of the nucleosome sequence data set

For this study, chromatins were prepared by reconstitution using an equimolar combination (mixture) of plasmid DNA molecules and a variety of core histone preparations. The recombinant DNA mixture comprised, in equimolar amounts, (i) a 10,300-bp segment of human DNA containing a version of the insulin gene with a class I polymorphic region (ILPR) just upstream of its promoter (Phins), (ii) a 10,841-bp segment of sheep DNA containing the β-lactoglobulin gene (BLG), (iii) a 4860-bp segment of *Drosophila simulans* DNA carrying a copy of the *Mos1* transposon (Mos1) and (iv) a 13,626-bp segment of *Saccharomyces cerevisiae* DNA incorporating a late-firing replication origin (YRO).

In separate reconstitutions, five different preparations of core histones were employed. All of these were recombinant proteins except for the chicken histones that were isolated from mature erythrocytes. Recombinant histones were prepared using frog (*Xenopus laevis*) and yeast (*S. cerevisiae*) histone genes and were purified as previously described [24]. In one preparation of yeast histones, H3 was replaced by the centromere-specific histone variant, Cse4. Recombinant histones were purified as octamers by size-exclusion chromatography. At this stage in the preparation of the canonical yeast histone octamer, H3/H4 tetramers and H2A/H2B dimers were also isolated. Consequently, we were able to form yeast chromatin in two ways: either by reconstitution with the purified histone octamer or by reconstitution with a mixture of tetramers and dimers. To form an octamer from the yeast dimers and tetramers, we added together aliquots in a 2:1 molar ratio prior to reconstitution.

**Fig. 1:**
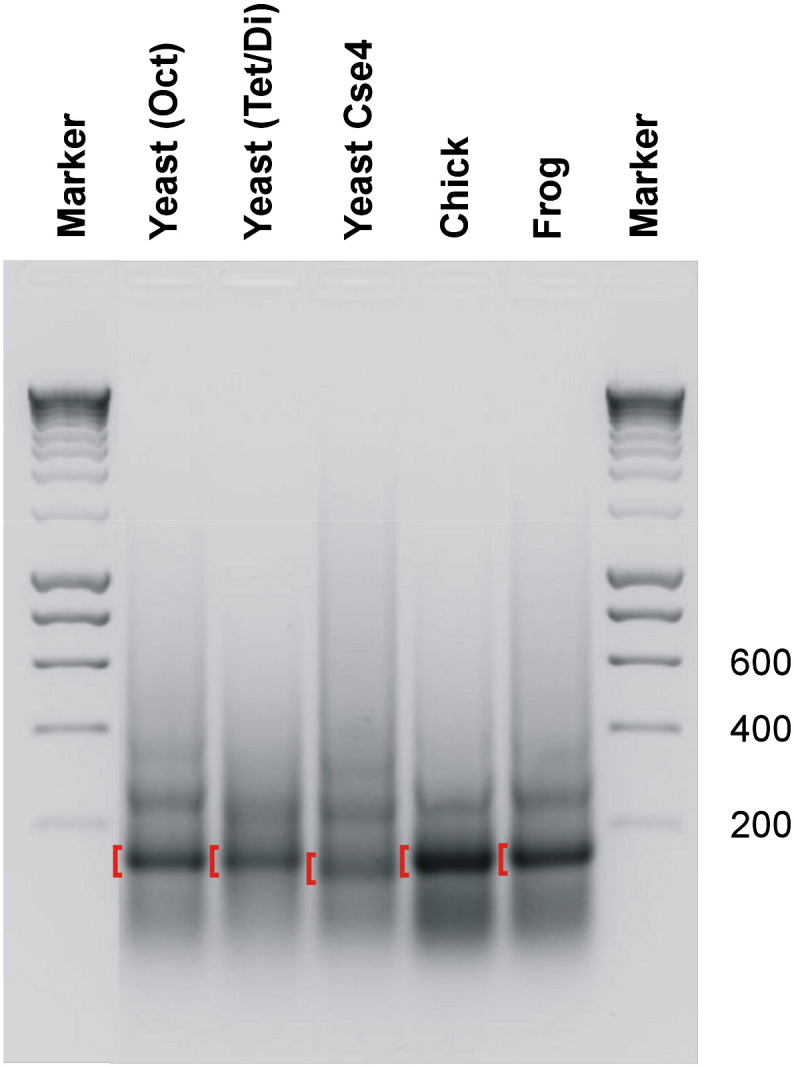
Nucleosomal DNA preparation. DNA recovered after micrococcal nuclease digestion of reconstitutes prepared with five different types of histone preparation was fractionated by agarose gel electrophoresis. DNAs cut from the gel are indicated by the red brackets. Selected marker DNA sizes are indicated.

The DNA mixture was reconstituted with limiting amounts of the different core histones by salt gradient dialysis [8,23]. Reconstituted chromatins were digested with micrococcal nuclease to produce a population of mononucleosomes. DNA was isolated from these particles, and fragments of approximately 150 bp were purified by gel electrophoresis. At this stage, it was noted that the monomer DNA fragment arising from digests of reconstitutes prepared with the Cse4-containing octamer was consistently smaller than monomer DNAs from the other digests (Fig. 1; Supplementary Fig. 1).

Using the selected monomer DNAs (Supplementary Fig. 1), we determined the locations and relative abundance of the preferred sites of histone octamer positioning on the different DNAs by high-throughput sequencing. Illumina paired-end sequencing provided, on average, a total of 16.2(± 1.5) × 10 [6] pairs of reads per sample (reconstitute) of which 70.4(± 1.5)% were aligned with high confidence to the four separate reference sequences. The bulk of the remaining reads aligned to plasmid vector sequences and the total alignment rate was greater than 95%.

**Fig. 2:**
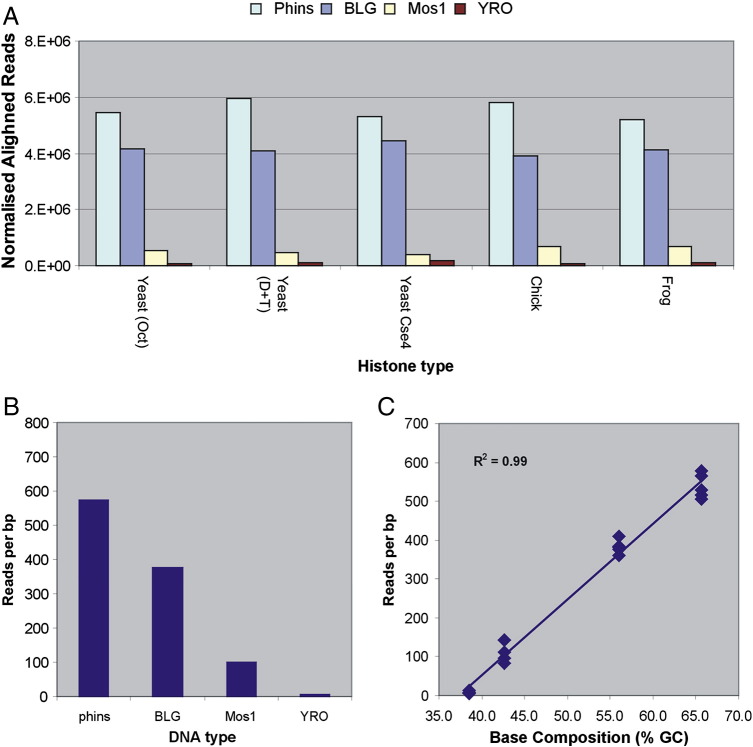
Sequence read numbers. (a) Numbers of paired-end sequence reads mapped onto the four DNA sequences are presented as a function of the type of core histone used for reconstitution. The total number of reads for each reconstitute was normalized to 15 × 10^6^. (b) The number of sequence reads per base pair for each of the DNA sequences averaged over the five types of reconstitute. (c) The number of sequence reads per base pair, for all five types of reconstitute, is plotted as a function of the G + C content of each DNA sequence. The correlation coefficient (*R*^2^) for the data is shown.

The number of reads that aligned to each DNA sequence (excluding the vector) was biased with respect to the source of genomic DNA and was strongly correlated with the base composition (G + C content) of the sequence (Fig. 2). Thus, the number of reads mapping to the human Phins (65.6% GC) and sheep BLG (56.0% GC) sequences were consistently greater than expected (observed to expected ratios of 2.17 and 1.43, respectively) whereas the reads mapping to the drosophila Mos1 (42.6% GC) and yeast YRO (38.5% GC) sequences were notably underrepresented, particularly in the case of the yeast DNA (0.37 and 0.03, respectively). The source of histones used to prepare the chromatins had relatively little influence upon the read number bias and essentially the same pattern was observed in all reconstitutes (Fig. 2a). Given that sets of DNAs were reconstituted, digested and gel purified as a mixture, the differences in read numbers cannot be attributed to experimental variation in these aspects of the processing procedure. Although the sequencing process is known to be somewhat biased, the underrepresentation of particular sequences tends to occur in a unimodal manner such that both AT- and GC-rich DNA sequences are underrepresented [25,26]. Furthermore, the extent of the bias, over the GC range of the DNAs used in this study, is generally no more than 5-fold [25,26], which is substantially less than the 2 order of magnitude difference we observe (Fig. 2).

**Fig. 3:**
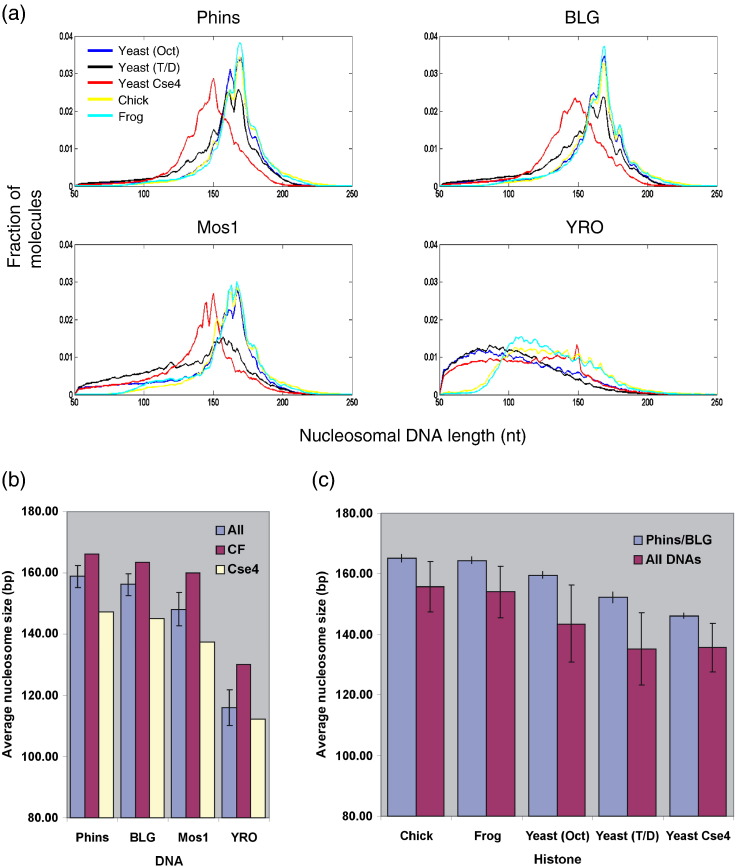
Size distributions of the histone octamer binding sites. (a) The distributions of nucleosomal DNA lengths, obtained from paired-end sequencing of the DNAs recovered from the five different core histone reconstitutes (color coded) for each of the four DNA sequences, are shown. Numbers of molecules are presented as a fraction of the total number of molecules indicated by paired-end reads that aligned to each DNA in each reconstitute. (b) Average lengths of nucleosomal DNAs recovered from selected reconstitutes [all reconstitutes, chicken and frog reconstitutes averaged (CF) and Cse4 reconstitute] formed on each type DNA. (c) Average lengths of nucleosomal DNAs recovered from Phins and BLG or from all DNAs for all five histone types.

The size distributions of the molecules identified by the paired-end reads that aligned to each of the four reference sequences reveal the lengths of DNA protected by the various histone octamers during nuclease digestion (Fig. 3; Supplementary Fig. 2). In most cases, the distributions fall largely within the expected size range 145–175 nt [8] and the DNA lengths tend to be quantized with clear peaks at ~ 149, ~ 159 and ~ 168 nt, as previously observed [8]. However, the size distributions arising from some of the samples were unusual. For example, the sizes of the protected fragments arising from chromatin formed on the YRO DNA were consistently shorter than typical nucleosome size. In general, these fragments displayed a broad size distribution with a mean length of around 110 nt (Fig. 3a and b) although both the distribution and average size varied somewhat with the type of histone octamer used for reconstitution (Fig. 3a; Supplementary Fig. 2). Thus, protected fragments from YRO were generally longer in reconstitutes prepared with chicken or frog histones when compared with any of the reconstitutes made with yeast histones. For the reconstitute prepared with a tetramer/dimer mixture of yeast histones, the sizes of protected fragments arising from the Mos1 DNA were also somewhat shorter than normal although the reduction in fragment size was not as great as that seen on YRO, and for each of the other four reconstitutes, protected fragment lengths arising from Mos1 were essentially indistinguishable from those obtained from BLG and Phins (Fig. 3). Finally, on all of the DNA sequences examined, the yeast Cse4-containing octamer consistently protected nucleosomal fragments that were about 20 bp shorter than those obtained with the chicken or frog histones (Fig. 3a and b). This observation is consistent with the reduced size of the bulk monomer DNAs isolated from chromatin reconstituted with Cse4-containing yeast histones (Fig. 1; Supplementary Fig. 1). A short (~ 125 bp) DNA length associated with the Cse4-containing core particle has been observed previously [27] and is proposed to arise from a weakness in the association of terminal DNA with the histone octamer [28].

At first sight, the short nucleosomal DNA derived from YRO reconstitutes would appear to be at odds with the nucleosomal DNA sizes indicated by agarose gel analysis (Fig. 1; Supplementary Fig. 1). However, given that the number of reads derived from YRO constitute only a small fraction of the total reads from any reconstitute (Fig. 2), the nucleosomal DNA size distributions suggested by gel analysis are in fact more representative of the other DNAs present (mainly Phins and BLG). Presumably, the generous size of the gel slice taken when isolating the monomer DNAs allows the capture of fragments as small as those seen in the YRO analysis.

### Generation and properties of histone octamer positioning maps

We have used two methods to represent the histone octamer binding sites (nucleosome positioning sites) identified on the DNAs used for reconstitution. Firstly, the data are simply presented in terms of the coverage attained through sequencing. Secondly, we have also employed a procedure previously developed [8] to identify positioning site dyads from paired-end sequencing data.

**Fig. 4:**
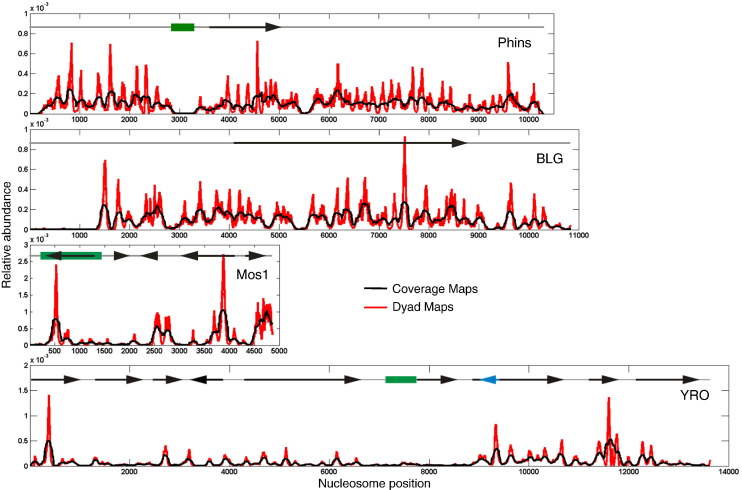
Core histone octamer positioning on genomic DNA sequences. The locations and relative abundance of frog histone octamer binding sites on each genomic DNA are presented in terms of (i) sequencing coverage (black) and (ii) calculated nucleosome dyads (red; see Methods). The maps have been adjusted so that the total signal for each map is normalized (to unity). Schematic representations of the gene structures (transcribed sequences) within each of the genomic regions are indicated (arrows; blue for the overlapping gene on the opposite strand on YRO) and the locations of the ILPR (Phins), the Mos1 transposon (Mos1) and the late-firing replication origin (YRO) are identified by green rectangles.

Frog histone octamer positioning maps for Phins, BLG, Mos1 and YRO are shown in Fig. 4. The maps have been adjusted so that the total signal for each map is normalized (to unity). Consequently, the relative intensity of histone octamer binding sites on the separate maps is not directly comparable from a quantitative point of view. Another set of maps, in which the total signal for each profile has been corrected for the number of sequence reads that aligned to each sequence (Fig. 2) and is intended to allow a comparison of the relative affinity of the histone octamer for each of the four types of DNA, is presented in Supplementary Fig. 3.

In considering the general character of the four, octamer-positioning maps, those of Phins and BLG stand out from the other two in terms of the density of sites that are frequently occupied (high-affinity sites). Generally, the YRO and Mos1 sequences present a small number of strong positioning sites suggesting that most of these sequences display a relatively low affinity for the histone octamer.

The *in vitro* nucleosome maps for BLG, YRO and Phins can be compared to previously published, *in vivo* data. Thus, we have already established a relationship between *in vitro* and *in vivo* nucleosome positioning on BLG [8,29,30]. Similarly, our *in vitro* nucleosome positioning on the YRO plasmid shows a fairly strong correlation (*R* = 0.55) to positioning data collected *in vivo* for the yeast genome [3] (Supplementary Fig. 4). This is consistent with DNA sequence contributing to the positioning at a subset of the locations observed *in vivo*. However, a comparison of our results for the insulin-containing Phins plasmid with positioning data obtained from primary human cells [31] is less informative. Due to sequence differences (mainly indels) between our plasmid and their reference sequence, particularly in the polymorphic ILPR, it is difficult to justify a quantitative comparison. A superficial analysis suggests a fair degree of similarity (Supplementary Fig. 5).

The positioning map for the insulin gene sequence (Phins) has a gap in the region of the ILPR (Fig. 4; Supplementary Fig. 6). However, as Southern-blot analysis indicates that nucleosomes are effectively assembled at this location (Supplementary Fig. 7), sequences derived from the ILPR must be present in the DNA population subject to high-throughput sequencing. There are two reasons why it may be difficult to map nucleosome positioning in the ILPR. Firstly, as it is composed of tandem repeats of a 14-bp sequence (consensus, TSYGGGGACAGGGG) [32], paired-end reads derived from the ILPR cannot be uniquely aligned at this site [33]. These reads, the majority of which align at multiple locations (multi-reads), are not included in the file used to determine our high-resolution nucleosome positioning profiles. Secondly, we have shown previously that the sequence repeats in the ILPR can adopt quadruplex structures [34]. As these are known to effectively inhibit the elongation of DNA polymerase [35], they may contribute to the failure to detect sequence reads mapping to the ILPR by directly inhibiting the sequencing procedure. In support of this argument, we note that (i) the number of multi-reads that align to the ILPR constitute only about 11% of what would be expected on the basis of the size and GC content of the ILPR and (ii) that the reads that do align here show a pronounced strand bias with < 10% aligning to the coding strand of the ILPR that contains the G-rich sequence (TSYGGGGACAGGGG) with the potential to form a quadruplex structure (Supplementary Table 1).

Histone octamer positioning profiles for the Phins, BLG, Mos1 and YRO sequences, presented as a function of histone type used for reconstitution, are shown in Supplementary Fig. 8a–d. A visual comparison of the maps for each DNA suggests a reasonably high degree of similarity, except in one case. Generally, it is clear that the chicken, frog and yeast histones all bind to the same spectrum of positioning sites although careful inspection indicates that the relative abundance with which particular positioning sites are occupied does vary with histone type. At higher resolution, the extent of these quantitative differences in site occupancy is more apparent (Supplementary Fig. 9). Thus, on particular regions of sequence, it would appear that the equilibrium distribution of binding site occupancy can be somewhat sensitive to the type of core histone used for reconstitution.

On the yeast sequence (YRO), histone octamer type-dependent differences in the binding site occupancy profiles appear to be more pronounced (Supplementary Fig. 8d). Here, visual inspection shows that the binding site profiles obtained with the canonical yeast octamer or yeast tetramer/dimer mixture are very alike. The binding profiles obtained with the chicken and frog histones are also very similar to each other but are clearly distinct from the canonical yeast histone profiles. Finally, the map obtained with the yeast Cse4 histones appears to be different to all the other profiles. However, although these differences between the YRO profiles are easily identified, especially when contrasted with the similarity of the corresponding profiles on the other three DNAs, there is nevertheless much in common between the five YRO maps.

To compare the various histone octamer positioning site data sets more rigorously, we have employed scatter plots. For this purpose, we converted the occupancy profiles to indicate the relative free energy (Δ*G*^0^) of association of the histones with the DNA using the following equation:

$$\Delta G_{i}^{0}=-\mathrm{RT}\ln\left(I_{i}\right)$$

where *R* is the molar gas constant, *T* is the temperature in Kelvin and *I_i_* denotes the occupancy level of positioning site *i*. *I_i_* values at each nucleotide were derived from the dyad nucleosome positioning maps.

**Fig. 5:**
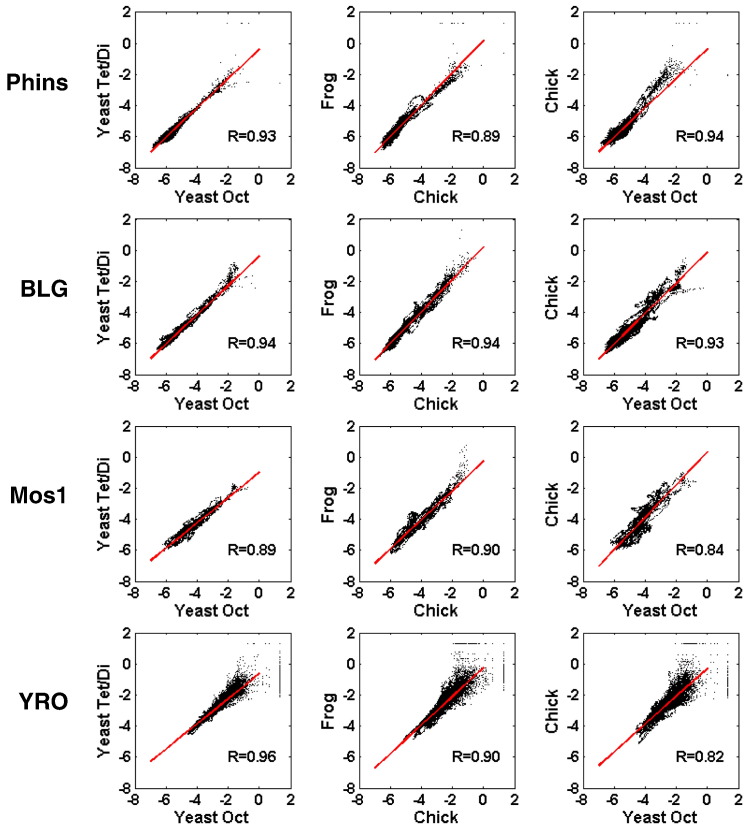
Relationships between histone octamer positioning site affinity maps. Scatter plots of the relative free-energy values (Δ*G*^0^) of binding sites, derived from the dyad profiles for selected pairs of histone type maps, are presented. *R* values derived from linear regression analysis (red line) are shown on each panel. In the context of the average for the four DNAs, the panels have been ranked from the highest (left) to the lowest (right) with respect to *R*.

**Fig. 6:**
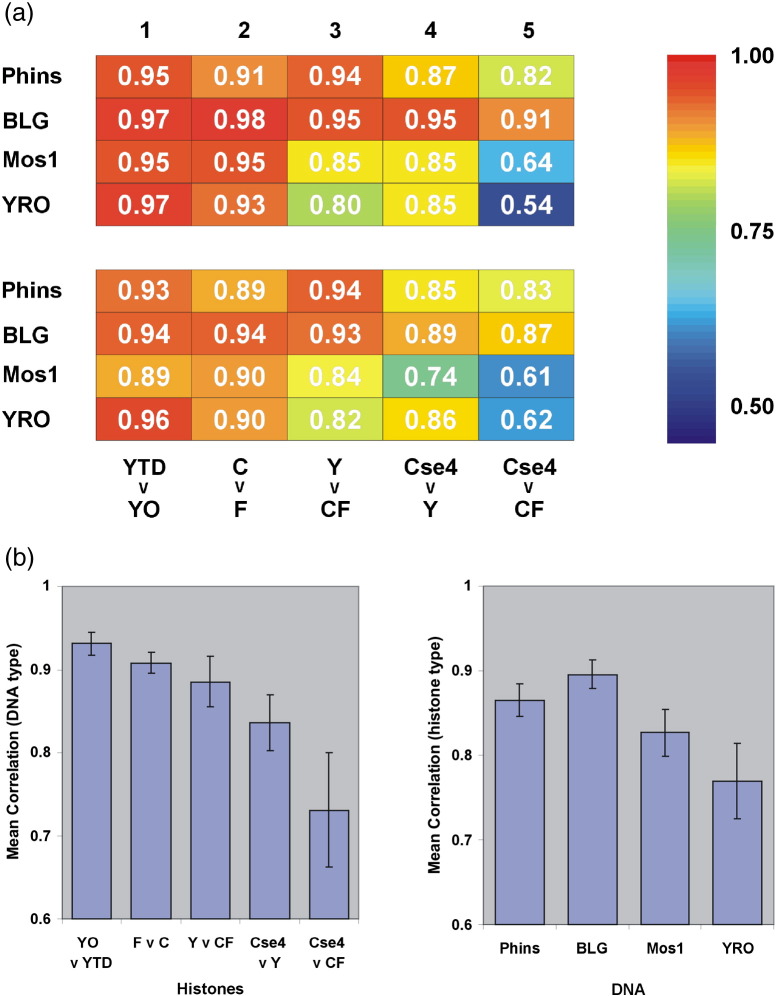
Relationships between histone octamer positioning site affinity maps. (a) *R* values, derived from linear regression analysis of scatter plots of the relative free-energy values (Δ*G*^0^) of positioning sites, for the coverage (top panel) and dyad (lower panel) profiles, are presented in color-coded format. YTD, yeast (tetramer/dimer); YO, yeast octamer; C, chick; F, frog; Y, an average of the yeast octamer and the yeast tetramer/dimer data; CF, an average of the chicken and frog data; Cse4, Cse4 yeast octamer. (b) The correlation values (*R*) from the analysis of the dyad profiles [lower panel in (a)] were used to determine the mean correlations for all analyses of selected histone-type comparisons (left panel) and for all analyses of each DNA type (right panel).

A selection of the scatter plots is shown in Fig. 5, and a more comprehensive summary of the quality of all scatter plot correlations is presented in Fig. 6. Overall, this analysis confirms the strong relationship between the maps produced with different types of core histones although the correlation coefficients (*R*) clearly indicate that the relationships between the various pairs of maps do vary significantly (Figs. 5 and 6). The following points are noteworthy:

• The maps obtained with either the purified octamer or the tetramer/dimer mixture of the yeast histones are essentially indistinguishable, a feature that is independent of the DNA type (Fig. 6a, column 1).
• The maps obtained with the (native) chicken or (recombinant) frog histones are extremely similar, a feature that is independent of the DNA type (Fig. 6a, column 2).
• The maps obtained with the yeast histones or the chick/frog histones are extremely similar when Phins or BLG is the DNA substrate but are less alike when Mos1 or YRO is the DNA substrate (Fig. 6a, column 3).
• The maps obtained with Cse4-containing yeast histones are well correlated to the canonical yeast histone maps and to the chicken/frog maps when Phins or BLG is the DNA substrate. However, on Mos1 or YRO DNA, there is a notable reduction in the correlation between the Cse4-containing yeast histone maps and the chicken/frog maps (Fig. 6a, columns 4 and 5).

A final insight into the effect of histone type (and DNA type) upon nucleosome positioning is presented in Fig. 6b. Here, where we have averaged the correlations between maps for (i) selected histone-type comparisons and for (ii) DNA type, we can see that the relationship between binding site maps is sensitive not only to the nature of the histones being compared but also to the sequence of the DNA upon which they are being compared.

### Some sequence properties of the nucleosomal data sets

**Fig. 7:**
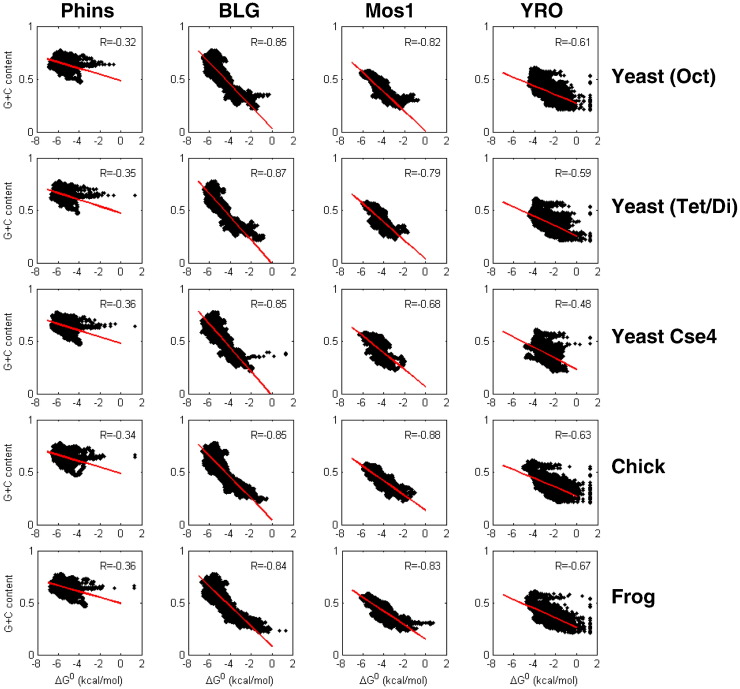
Relationships between histone octamer binding site affinity maps and DNA sequence. Scatter plots of the relative free-energy values (Δ*G*^0^) of the binding sites identified in the dyad profiles, measured on each DNA reconstituted with the indicated type of core histone, and the base composition (G + C content) of the binding site, measured in a 111-bp window centered on the dyad of the site, are shown. For each scatter plot, the correlation (*R*) is indicated.

It is well established that the G + C content of DNA has a major influence in determining the affinity of the histone octamer for different positioning sites [6,36–39]. This relationship may reflect the relatively flexible nature of the GC base pair step and consequently the lower energetic cost of wrapping G + C-rich DNA sequences onto the histone octamer [6]. It follows that the relationship might also be dependent on (i) the precise geometry of the charged surface of the octamer and (ii) the stability of the octamer, parameters that might vary with octamer type. Consequently, we have examined this property in some detail to establish whether it does vary as a function of histone octamer source. Each of the four DNA sequences was scanned for G + C content using a variety of window sizes to generate base composition profiles. These were then compared, by scatter plot analysis, with the relative free-energy (Δ*G*^0^) profiles of the corresponding histone octamer positioning maps. The results (Fig. 7) indicate a clear relationship between the G + C content of binding sites and their relative affinity for the histone octamer. The correlation is particularly striking for analyses carried out on the BLG and Mos1 DNAs where the average *R* values derived from linear regression of these data sets are − 0.86 (± 0.01) and − 0.80 (± 0.03), respectively (Fig. 7). The narrow range of *R* values within each of these sets indicates that the relationship is relatively independent of the histone type employed for reconstitution. Analysis of the corresponding plots on the YRO and Phins DNAs shows that they are less well correlated, with average *R* values of − 0.54 (± 0.06) and − 0.37 (± 0.02), respectively. On YRO, the data for the Cse4 octamer are notably less well correlated. If, for this DNA, a comparison is restricted to the canonical yeast, chicken and frog data sets alone (omitting the Cse4 data), the average *R* value improves to − 0.63 (± 0.02).

**Fig. 8:**
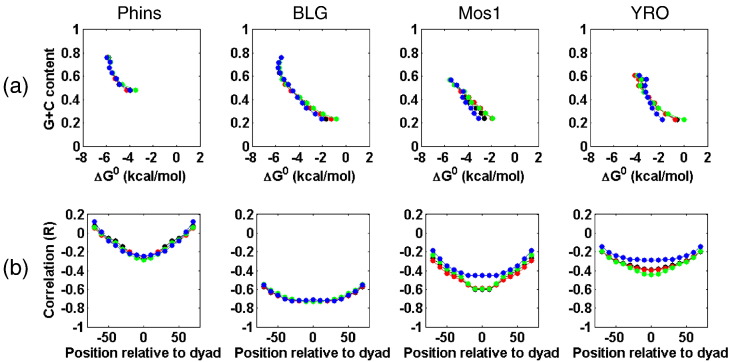
Relationships between histone octamer binding site affinity maps and DNA sequence. (a) The relationship between the average relative free energy (Δ*G*^0^) of binding sites, derived from the dyad profiles, binned in respect of G + C content (5% bin size), shown for analyses using yeast histones (black), Cse4 histones (blue), chicken histones (red) and frog histones (green), is presented for each of the four DNA types. (b) The correlation (*R*) between the relative free-energy values (Δ*G*^0^) of binding sites and the G + C content within defined regions (windows) of the binding sites are presented as a function of the location of the base composition windows relative to the dyad of the binding site. G + C content profiles were generated for each sequence with (i) a central 11-bp window and then with (ii) a set of pairs of 10-bp windows centered on and straddling the central 11-bp window and moving out from the center in 10-bp steps. Analyses for reconstitutes prepared with yeast histones (black), Cse4 histones (blue), chicken histones (red) and frog histones (green) are shown for each of the four DNA types.

In Fig. 8, the association between G + C content of binding sites and their relative affinity for the yeast, Cse4, chicken and frog octamer is presented in an alternative form. Here, the data have been binned in respect of their G + C content before calculating the average free energy (Δ*G*^0^) of points falling within each bin. Plots of the binned and averaged data permit a simple visual comparison with regard to the influence of histone octamer type (Fig. 8a) on the G + C/binding site strength relationship.

This analysis confirms that G + C has a major influence on the affinity of DNA for the histone octamer under our non-saturating, competitive conditions. Generally, the strength of a binding site is proportional to its G + C content, a relationship that, for BLG and Mos1 in particular, is relatively linear for G + C contents between about 25% and 60%. For these two DNAs, the slope of the plots in this G + C range, for the yeast, chicken and frog histones, indicates that, on average, an ~ 10% increase in G + C content results in an improvement in binding affinity equivalent to a decrease in Δ*G*^0^ of 1 kcal/mol (Figs. 7 and 8a; Supplementary Fig. 11). However, in this context, it should be noted that, for the yeast Cse4 reconstitutes, the corresponding increase in G + C content required for the same change in free energy is increased on Mos1 (~ 15%) and almost doubled (~ 20%) on YRO (Fig. 8a; Supplementary Fig. 11), indicating that, by this criteria, the Cse4 octamer can be distinguished from the other histones.

Sites with very low or very high G + C contents tend to depart from this (linear) relationship, and both of these conditions tend to have a negative influence upon the strength of binding (Fig. 8a). At particularly low G + C (< 25%), this behavior is likely to not only reflect the paucity of G/C but will also result partly from an increased occurrence of tracts of poly(dA:dT) that are known to be refractory to histone octamer binding [5,10]. On the other hand, the data obtained from analysis on Phins and BLG (Fig. 8a) show that, as the G + C content rises above 60%, the binding affinity of sites stops increasing and, in the case of BLG, begins to fall, although sites with a G + C content of ~ 80% still remain among the very highest affinity binding sites.

It is noteworthy that, at any particular value of G + C content, the scatter plots display a broad range of Δ*G*^0^ values (Fig. 7) approximating to ~ 2–3 kcal/mol. Thus, although G + C content may play a key role in determining the affinity of a positioning site for the histone octamer, other aspects of sequence arrangement clearly remain a significant influence. At very high values of G + C and for DNA molecules, or regions of molecules, with a relatively narrow range in G + C content, these other aspects of sequence organization will have a proportionately greater influence in determining the relative affinity of a binding site for the histone octamer. It is for these reasons that, in spite of having a high density of high-affinity binding sites, the correlation between G + C content and binding site strength is comparatively poor for Phins (Fig. 7).

We have also analyzed the histone octamer positioning sites to determine whether the preference for G + C-rich sequence is a general property of the entire (147 bp) binding site or whether it tends to be focused with respect to the center or dyad of the site. For this analysis, we constructed base composition profiles for each sequence using (i) a central 11-bp window and then (ii) a set of pairs of 10-bp windows centered on and straddling the central 11-bp window and moving out from the center in 10-bp steps. These base composition profiles were then compared, by scatter plot analysis, with the relative free-energy (Δ*G*^0^) profiles of the histone octamer positioning sites, and the correlations between the various data sets were determined. These resulting *R* values were then plotted as a function of the location of the base composition windows relative to the center of each binding site. The results shown in Fig. 8b show that, for all DNAs, the quality of the relationship between G + C content and binding site strength has a distinctive distribution with respect to the center of the octamer binding site. Generally, the best correlation is to sequences closer to the dyad. On moving toward the edges of the binding site, the correlation diminishes. This result suggests that G + C content closer to the dyad has a greater influence on the strength of the resulting binding site than the sequences toward the ends of the site. Of course, a complementary perspective is that the binding site strength is inversely proportional to A + T content and that the tendency of A/T-rich sequence tracts to be restricted to the boundaries of the nucleosome binding site contributes to the characteristic bell-shaped profiles seen in Fig. 8b. In respect of this property, the data obtained with yeast, chicken and frog histone (octamers) appear to be indistinguishable, on all four DNA types. However, it is again clear that, for this type of comparison, on the more A + T-rich DNAs (Mos1 and YRO), the yeast Cse4 octamer appears distinct from the other histones (Fig. 8b).

**Fig. 9:**
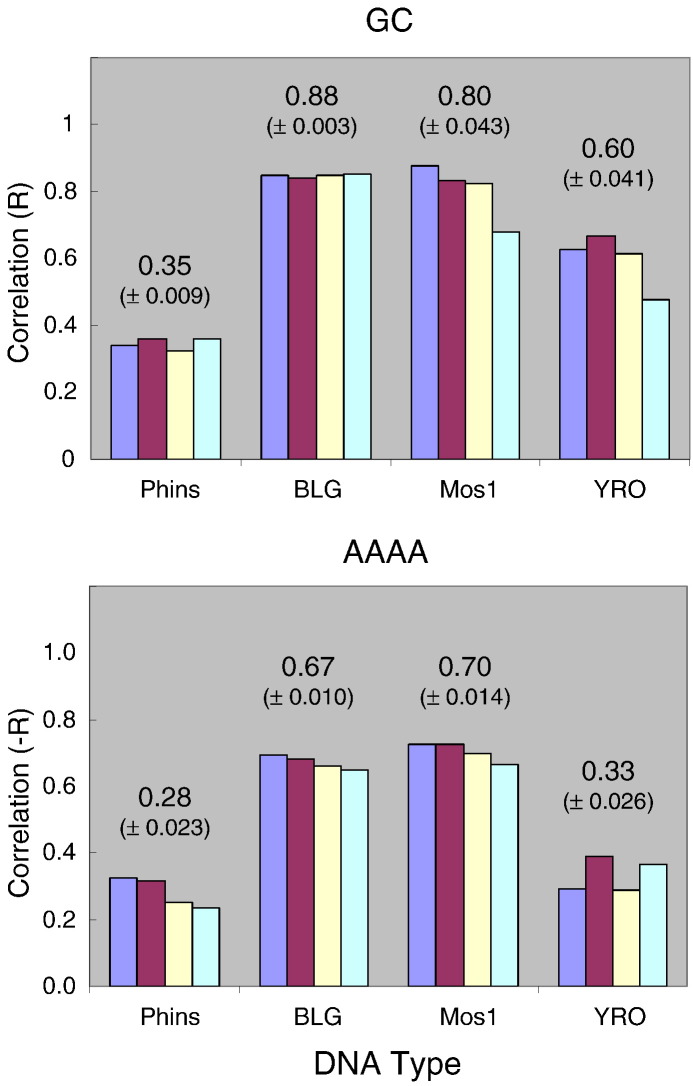
Relationships between histone octamer binding site affinity maps and DNA sequence. The correlation (*R*) between the relative free energy (Δ*G*^0^) of binding sites, derived from the dyad profiles, and G + C content (upper graph) or AAAA frequency (lower graph) for data derived from reconstitutes prepared with chicken (blue), frog (red), yeast (yellow) and Cse4 (cyan) histones are presented as a function of DNA type. The average correlation (*R*) and standard error for each group is shown.

Although G + C content has a substantial influence in determining the relative affinity of a DNA for the histone octamer, the frequency of stretches of poly-A within the binding site also makes a significant contribution [3,10,40]. Indeed, it is argued that these two parameters alone are accurate predictors of nucleosome positioning *in vitro* [40]. Poly-A sequences are thought to resist the bending required to form a nucleosome [10,41] and are therefore relatively resistant to histone octamer binding. We have also examined the relationship between our nucleosome positioning profiles and the occurrence of poly-A tracts within binding sites. Each of the DNA sequences was scanned to determine the frequency of AAAA within a 147-bp window, and these profiles were then compared, by scatter plot analysis, with the relative free-energy (Δ*G*^0^) profiles of the corresponding histone octamer positioning maps. The results (Fig. 9; Supplementary Fig. 10) clearly indicate that this sequence motif has a negative influence upon histone octamer binding. Again, as seen in the G + C study, the correlations are particularly strong for the analyses carried out on the BLG (12.5 occurrences of AAAA per kilobase pair) and Mos1 (35.2 occurrences per kilobase pair) DNAs. For the Phins DNA, the relatively low correlations are likely to reflect the very low abundance of AAAA tracts in this particularly G + C-rich DNA (2.5 occurrences per kilobase pair). However, one cannot use this argument to explain the poor correlations between the frequency of AAAA and histone binding strengths on YRO as the density of the motif on this sequence is much higher (27.4 occurrences per kilobase pair) and more than twice that found on BLG. Although it is evident that poly-A tracts influence histone octamer binding, it would appear that this influence is dependent, not surprisingly, on other features of local sequence composition or organization. For example, our limited data might suggest that AAAA tracts are more effective in influencing nucleosome positioning in regions where the average AT (or GC) content is relatively neutral (BLG and Mos1) but are less effective in more extreme sequence environments (Phins and YRO).

We have also surveyed the correlations between our nucleosome positioning maps and the frequency of each dinucleotide, trinucleotide and tetranucleotide sequence motif within the binding sites. A summary of this analysis is shown in Supplementary Table 2. As with the AAAA data, there is little indication to suggest that the influence that particular sequence motifs have upon histone binding varies with the type of octamer (Fig. 9; Supplementary Table 2).

## Discussion

In this study, we have investigated the binding specificity of four types of core histone octamer to a variety of DNA sequences, *in vitro*. The aim was to determine whether the well-established binding preferences that histones show for different DNA sequences is influenced by the amino acid variation inherent in the core histones from different species and to ascertain whether this constitutes a factor that might influence nucleosome positioning and, consequently, *in vivo* chromatin structure. The results could also have relevance to the interpretation of comparisons made between whole genome nucleosome positioning studies carried out on cells derived from a wide range of evolutionarily diverse organisms. Although it is conceivable that a limited form of this type of analysis could be carried out *in vivo*, by replacing the histone genes in yeast, for example, the experiment is most effectively carried out *in vitro* where the properties of the histones can be investigated in the absence of the many other factors that modulate nucleosome positioning *in vivo*.

The process we employed in our study involved the reconstitution of core histone octamers onto a mixed population of plasmid DNAs. Nucleosomal DNA molecules, recovered from reconstituted chromatin after nuclease digestion, were then sequenced to identify histone octamer positioning sites. In terms of methodology, the only variable was the source of histone octamer used for reconstitution. Consequently, we anticipated that it should be a relatively straightforward task to identify whether there is a histone octamer-specific influence upon nucleosome positioning. However, it is evident from our results that the considerable influence of DNA sequence upon the process is a complicating factor. During reconstitution, the histones display a particularly strong preference to associate with sequences that are rich in G + C (Fig. 2). Consequently, the final density of nucleosomes on the reconstituted plasmids are likely be higher for G + C-rich sequences (Phins and BLG) and lower for A + T-rich sequences (Mos1 and YRO) than that dictated by the histone-to-DNA ratio used for reconstitution (approximately one octamer per 500 bp).

The sequence-directed bias in the density of histone octamers assembled onto DNA during reconstitution will influence the way micrococcal nuclease digests the chromatins formed on the different DNAs. One might expect, for example, that the low-density chromatin formed on YRO would be, relative to the other chromatins in the sample, more rapidly digested and probably more susceptible to intranucleosomal cleavage. Furthermore, the preference micrococcal nuclease shows that AT-rich DNA [42,43] will act to enhance these features. The short size distribution of the nucleosomal DNA fragments recovered from YRO reconstitutes (Fig. 3) is entirely consistent with this interpretation. Interestingly, it is this feature of the process that identifies a significant difference between the higher eukaryotic histones (chicken or frog) when they are compared to the yeast histones as the former are clearly more capable—although still not completely competent—in protecting YRO-derived nucleosomal DNAs from intranucleosomal cleavage (YRO panel, Fig. 3a). With the exception of a single Mos1 sample (yeast tetramer/dimer), all other nucleosomal DNA samples are of typical size and distribution, except those arising from the Cse4 reconstitutes. Here, however, the ~ 20-bp reduction in DNA size associated with these nucleosomes (Fig. 3a and b) is clearly a *bone fide* property of the Cse4 octamer reflecting its relatively poor capacity to completely enfold a full length of nucleosomal DNA [27,28].

Our nucleosome dyad mapping procedure, which works by identifying and averaging the centers of a wide range of possible binding site sizes, based on the locations of their upstream and downstream ends, should be relatively insensitive to the size of the DNA fragments recovered after nuclease digestion, assuming that the trimming from a large (~ 190 bp) to a small (~ 110 bp) binding site occurs with equivalent likelihood at the upstream or downstream end of the protected, nucleosomal DNA fragment. Two further points support this contention. Firstly, in a previous study [23], where we compared caspase-activated DNase with micrococcal nuclease as tools for mapping nucleosome positioning, we used a mixture of BLG and YRO for reconstitution. In that study, the YRO nucleosomal DNA fragments recovered were less degraded (than in the current study) and closer to full-length nucleosomal DNA—although they were still shorter than fragments recovered from BLG. The number of YRO reads were also substantially less than for BLG—but not as pronounced as in the current work. Although these differences may partly reflect experimental variation, they may indicate that the change in the competitive reconstitution conditions (particularly the inclusion of the very GC-rich Phins plasmid in the current study) shifts the equilibrium during reconstitution to the “detriment” of nucleosome assembly/stability on YRO, causing it to become more nuclease sensitive. However, and this is the essential point, a comparison of the YRO dyad maps from these two independent studies (CAD/MNase *versus* the current work) provides a correlation of 0.73 (*R*) suggesting that nucleosomal DNA size does not have a pronounced effect on the mapping procedure. Secondly, as pointed out earlier, our current YRO nucleosome positioning maps are quite well correlated with *in vivo* data (Supplementary Fig. 4).

Consequently, the fact that binding sites recovered from Cse4 reconstitutes that are notably shorter than those from reconstitutes prepared with the other histone types will have limited impact when positioning site maps are compared. Similar arguments can be made when considering the influence of the short nucleosomal DNA lengths arising from YRO and Mos1 reconstitutes (Fig. 3).

The comparison we have made between chromatins formed with either a yeast histone octamer or a mixture of yeast histone H3/H4 tetramers and H2A/H2B dimers serves an important purpose. We had assumed that this difference in procedure would not be an issue given that the pathway followed in the salt-dialysis-based assembly process involves, firstly, the binding of the H3/H4 tetramer to the DNA followed, at a lower salt concentration, by the binding of H2A/H2B dimers [44]. This assumption appears to be fully justified as chromatins formed with yeast histones, added as either an octamer or tetramers and dimers, are almost indistinguishable in terms of nuclease sensitivity (Fig. 1) and nucleosome positioning (Fig. 6). Consequently, this comparison not only serves as an indicator for the reproducibility of the method but also provides a benchmark correlation against which other histone-type comparison can be judged.

There is little evidence from our study to suggest that chicken and frog histone octamers can be distinguished in terms of their nucleosome positioning properties, irrespective of the DNA substrate. Although some minor differences could be of significance, they may, given the relative complexity of the assay, simply reflect experimental variation. It is worth noting that, as the chicken histones were prepared from adult erythrocytes, any difference arising from the presence of minor amounts of core histone variants or post-translational modifications do not appear to be a significant influence.

In contrast, it would appear that nucleosome positioning adopted by the canonical yeast histone octamer can be distinguished from that of frog or chicken histones, but only when measured on the relatively A + T-rich Mos1 or YRO DNA substrates. On the G + C-rich Phins and BLG DNAs, a significant difference is not detected (Fig. 6; Supplementary Fig. 8a–d). Although this interpretation may be complicated in the case of the measurements made on YRO, where the nucleosomal DNA lengths not only are shorter than usual but also display a clear dependence on histone type, this concern does not apply to measurements made on Mos1 where the size distribution of nucleosomal DNAs obtained with chicken, frog and canonical yeast histones are essentially the same (Fig. 3).

Although histones are highly conserved, of the three species examined in this study, the yeast histones are the most divergent [45]. Furthermore, although high-resolution analysis of core particles formed with chicken [46], frog [16] and yeast [18] histones indicates a highly conserved structure for the protein octamer, the complex containing yeast histones may be relatively less stable. A number of studies have previously suggested yeast histone-specific characteristics including a reduced capacity to reconstitute effectively with DNA [47] and an enhanced susceptibility of nucleosomes containing these histones to be unfolded [48,49].

The wild-type, recombinant yeast histones used in our study are notably distinguished from the other core histone types by virtue of their H3 complement. The sequence of H3 from *S. cerevisiae* identifies it as being equivalent to the replication-independent subtype, H3.3 [24,50,51], whereas for the recombinant frog histones, the H3 variant used was H3.1 [52,53]. In the case of the native chicken erythrocyte preparation, histone H3.3 is likely to comprise only a small fraction of the total H3 complement [54]. H3.3 tends to be found in transcriptionally active regions of the genome and is particularly abundant at regulatory or nucleosome-depleted regions of chromatin [55]. Furthermore, nucleosomes containing this H3 variant are prone to destabilization, particularly in combination with H2A.Z [55,56], and may be subject to altered nucleosome positioning [57]. In the light of these properties, the distinctive features of the yeast histone chromatin prepared in our study could be partly attributable to the presence of H3.3.

The other yeast core histone octamer type used in this study is also distinguished by the H3 it contains. Cse4 replaces H3 in the nucleosomes that occupy the centromeres on each budding yeast chromosome. Although, *in vivo*, the composition and structure of these specific nucleosomes remains a topic of vigorous debate [27,28,58], it appears that, *in vitro*, Cse4-containing histones form an octamer, rather than a tetramer, and that this octamer wraps the DNA in a conventional left-handed superhelical path when reconstituted into nucleosomes [27].

We expect that the nucleosomes formed with the yeast Cse4 octamer in our study conform, in general, to the conventional composition and structure. In spite of this, these histones form, in terms of nuclease digestion (Fig. 3) and nucleosome positioning (Figs. 6 and 8), the most distinctive set of nucleosomes analyzed in our study. For example, it is notable that when nucleosome positioning profiles are compared, (i) the Cse4 octamer is more like the yeast octamer than the chicken/frog octamer and that (ii) the difference between the Cse4 and the chicken/frog octamers is only revealed on the A + T-rich, Mos1 and YRO DNAs (column 5, Fig. 6a) suggesting a particular dependence upon the sequence (composition) of the substrate DNA. Given that a similar sequence dependence is required to distinguish the canonical yeast octamer from the chicken/frog octamer (column 3, Fig. 6a), it is tempting to suggest that yeast histones (both canonical and Cse4) are more adapted for an A + T-rich environment and that it is this property that distinguishes their nucleosome positioning properties.

On the more A + T-rich Mos1 and YRO DNAs, the Cse4 octamer is also distinct in terms of the relationship it exhibits between binding site strength and G + C content (Fig. 8a and b; Supplementary Fig. 11). These results indicate that, for sites with very low G + C content (very high A + T content), the reduction in binding strength seen for canonical yeast, chicken or frog histone octamers is not shared to the same extent by the Cse4 octamer. On YRO, for binding sites with the lowest G + C contents, the difference in free energy of binding (Δ*G*^0^) for the Cse4 data compared to the chicken, frog and yeast data is close to 2 kcal/mol (Fig. 8a). This may indicate that high A + T content and possibly poly-A tracts are less refractory to the binding of the Cse4 octamer than to the other octamer types. Although this might seem appropriate given that the native Cse4-containing nucleosome binding sites, CEN sites, are extremely A + T rich and contain a very high frequency of poly-A tracts [59], previous studies indicate that the Cse4 octamer shows no preference for such sequences [28].

In the context of the present study, it may be of relevance that, as the Cse4 octamer binds (~ 20 bp) less DNA in a nucleosome, it should be able to accommodate A + T-rich tracts toward its peripheries, whereas at equivalent positions, with the other octamer types, such sequences would be expected to have a destabilizing influence on nucleosome formation. This implies that simply because the Cse4 binding site is smaller, the spectrum of binding sites and their binding strengths will be different from those of an octamer that binds a canonically sized length of nucleosomal DNA. If this were to be the case, other factors that reduce the association of terminal DNA with the nucleosome structure, such as core histone acetylation, might have a similar effect upon nucleosome positioning.

Overall, there are indications from our study that the nucleosome positioning properties of different types of core histone octamer are distinguishable. However, this capacity seems to be dependent upon the type of DNA sequence onto which the histones are reconstituted. From our survey, it appears that differences between histone octamer types are more likely to be revealed and, consequently, to be of potential functional significance, on relatively AT-rich DNAs. In this context, it is tempting to speculate that the yeast genome with its distinctively short chromatin repeat length [44], demanding, perhaps, of a different form of folding into higher-order structure [60], may have evolved, in conjunction with the evolution of the yeast histones, a slightly modified scheme for identifying and utilizing nucleosome positioning sequences so as to help accommodate the particular packaging arrangements required for this highly transcribed, unicellular organism.

Finally, the work presented here does suggest that *in vitro* nucleosome positioning studies undertaken with genomic DNAs and, in particular, AT-rich genomic DNAs such as the yeast genome are probably best carried out with homologous histones.

## Methods

### DNA and histones

Four plasmid DNAs were employed: Phins321 comprised 10,300 bp of human DNA containing a version of the insulin gene that has a class I polymorphic region (ILPR) in its upstream promoter region and 4360 bp of plasmid vector [61]; pBLG (BLG) comprised 10,841 bp of ovine DNA containing the β-lactoglobulin gene and 2020 bp of plasmid vector [8]; p13 (YRO) comprised 13,626 bp of *S. cerevisiae* DNA containing a late-firing replication origin (ARS1413) and 6683 bp of plasmid vector [62]; *pMos1* (Mos1) comprised 4860 bp of *D. simulans* DNA containing a copy of the *Mos1* transposon and 3223 bp of plasmid vector [63].

Chicken erythrocyte core histones were prepared as previously described [29,64]. Recombinant *X. laevis* histones and *S. cerevisiae* histones, including Cse4, were expressed, purified, refolded and octamers isolated by size-exclusion chromatography [24,65]. During the preparation of the recombinant, canonical yeast histone octamer, H3:H4 tetramers and H2A:H2B dimers were also purified. During reconstitution, these dimers and tetramers were added together in a 2:1 molar ratio to form an octamer.

### Nucleosomal DNA preparation

An equimolar mixture of the Phins, BLG, Mos1 and YRO plasmids was reconstituted with core histones by salt gradient dialysis [29,64]. In independent experiments, chicken, frog and yeast histones were used to prepare reconstitutes. Three types of yeast histone reconstitutes were prepared. These were (i) yeast (octamer), (ii) yeast (tetramer/dimer mix) and (iii) yeast octamer in which H3 had been replaced with Cse4 [65]. Nucleosomal DNA was prepared from the reconstitutes as previously described [29]. Briefly, 25 μg of reconstituted chromatin was digested with 3 units of micrococcal nuclease (Worthington) for 30 min on ice, followed by 3 min at 37 °C. The resulting ~ 150-bp mononucleosome DNA fragments were purified after electrophoresis on a 1.5% agarose gels.

### DNA sequencing

Illumina/Solexa paired-end sequencing was undertaken by The Gene Pool at Edinburgh University†. Pre-processing involved blunt-ending of nucleosomal DNA by filling-in, adapter ligation and amplification by 18 cycles of PCR. Sequencing data and associated metadata have been deposited at the EBI Sequence Read Archive‡. Reference sequences for Phins, BLG, Mos1 and YRO are available at our Web site§.

### Alignment of sequence reads to the reference sequence

Paired-end sequence reads were aligned to the reference sequence using Bowtie∥ [66].

### Generation of nucleosome positioning maps

Nucleosome positioning data are presented in two ways. Coverage maps reflect the occurrence of each nucleotide of the mapped DNA in the fragments identified by the aligned sequence reads. Alternatively, maps depicting the dyads of histone octamer binding sites (positioning site dyads) have been generated essentially as previously described (***Method 2***) [8]. In this approach, a range of possible nucleosomal DNA lengths (all odd numbered lengths from 121 to 191) are considered. Each 5′ read count is paired with the appropriate 3′ read count for the nucleosome length being considered and the positioning site dyad established mid-way between these points. The amplitude of peak corresponding to a dyad is determined by the geometric mean (square root of the product) of the forward and reverse read counts. This method generates 36 maps, one for each nucleosome size being considered, which were summed for the final map. For comparison, all the above maps were normalized to unity.

## Footnotes

† http://genepool.bio.ed.ac.uk/

‡ http://www.ebi.ac.uk/ena/data/view/ERP002307

§ http://www.enps.bio.ed.ac.uk

∥ http://bowtie-bio.sourceforge.net/index.shtml
