# Supplementary material for: A Comparison of In Vitro Nucleosome Positioning Mapped with Chicken, Frog and a Variety of Yeast Core Histones
Source: J Mol Biol. 2013 Nov 15;425(22):4206–22. doi: 10.1016/j.jmb.2013.07.019 (PMC3899014; doi:10.1016/j.jmb.2013.07.019)
Supplement: Supplementary file 1 — Supplementary data [file mmc1.pdf]

Supplementary Figure and Table Legends

Figure 1

Nucleosomal DNA preparation. Agarose gel electrophoresis of purified, nucleosomal DNA fragments used for paired-end sequencing. Selected marker DNA sizes are indicated.

Figure 2

Size distributions of the histone octamer binding sites. The distributions of nucleosomal DNA lengths, derived from paired-end sequencing, for each genomic DNA sequence (colour coded), obtained from the five different core histone reconstitutes are shown. Numbers of molecules are presented as a fraction of the total number of molecules indicated by paired-end reads that aligned to each DNA in each reconstitute.

Figure 3

Core histone octamer positioning on genomic DNA sequences. The locations and relative abundance of frog histone octamer binding sites on each genomic DNA is presented in terms of (i) sequencing coverage (black) and (ii) calculated nucleosome dyads (red, see Methods). The maps have not been normalised in terms of total signal. Consequently the total signal for each map reflects the number of sequence reads that aligned to each sequence. This representation is intended to allow a comparison of the data in terms of the relative affinity of (frog) histone octamer for the four types of DNA.

Figure 4

Comparison of *in vitro* nucleosome positioning (coverage) on the YRO plasmid and *in vivo* nucleosome positioning on the corresponding region of the yeast genome. The *in vivo* data (Average normalized nucleosome occupancy) was taken from Kaplan et al (2008) *Nature* **458**, 362-366 and was downloaded from the Stanford University Saccharomyces cerevisiae genome browser.

http://browse.yeastgenome.org/fgb2/gbrowse/scgenome/?name=chrXIV:243179..256806

Figure 5

Comparison of *in vitro* nucleosome positioning (dyad) on the Phins plasmid and *in vivo* nucleosome positioning on the corresponding region of the human genome. The in vivo data (GM12878 cells) was taken from Valouev et al. (2011) *Nature* **474**, 516-520 and was downloaded from the UCSC Genome Browser.

Human chr11:2,175,709-2,186,039 - UCSC Genome Browser v284

Figure 6

Lack of sequence reads from the ILPR region of Phins. The number of paired-end reads that aligned to the region of the Phins DNA containing the polymorphic sequence (ILPR) is shown for DNA recovered from all five reconstitutes. The location of the ILPR sequence is indicated by the green rectangle. The transcription start site, and first exon, of the insulin gene are also shown.

Figure 7

Nucleosome formation on ILPR sequences. Agarose gel (EthBr) and Southern blot of DNA recovered from micrococcal nuclease digested chromatin formed on Phins. The plasmid DNA (Phins) was reconstituted with chicken histones and the resulting chromatin digested with micrococcal nuclease on a time course basis. Aliquots were removed at the indicated times and were fractionated on an agarose gel. For Southern blot analysis the DNA transferred to a filter was hybridised with a probe specific for the ILPR (^{32}P-5'-ACAGGGGTGTGGGG).Core histone octamer positioning on genomic DNA sequences. The locations and relative abundance of frog histone octamer binding sites on each genomic DNA is presented in terms of (i) sequencing coverage (black) and (ii) calculated nucleosome dyads (red, see Methods). The maps have not been normalised in terms of total signal. Consequently the total signal for each map reflects the number of sequence reads that aligned to each sequence. This representation is intended to allow a comparison of the data in terms of the relative affinity of (frog) histone octamer for the four types of DNA.

Figure 8a

Core histone octamer positioning on Phins DNA. The locations and relative abundance of histone binding sites on Phins genomic DNA, for each of the five types of reconstitute is presented in terms of (i) sequencing coverage (black) and (ii)

calculated nucleosome dyads (red, see Methods). The maps have been adjusted so that the total signal for each map is normalised (to unity).

Figure 8b

Core histone octamer positioning on BLG DNA. The locations and relative abundance of histone binding sites on BLG genomic DNA, for each of the five types of reconstitute is presented in terms of (i) sequencing coverage (black) and (ii) calculated nucleosome dyads (red, see Methods). The maps have been adjusted so that the total signal for each map is normalised (to unity).

Figure 8c

Core histone octamer positioning on Mos1 DNA. The locations and relative abundance of histone binding sites on Mos1 genomic DNA, for each of the five types of reconstitute is presented in terms of (i) sequencing coverage (black) and (ii) calculated nucleosome dyads (red, see Methods). The maps have been adjusted so that the total signal for each map is normalised (to unity).

Figure 8d

Core histone octamer positioning on YRO DNA. The locations and relative abundance of histone binding sites on YRO genomic DNA, for each of the five types of reconstitute is presented in terms of (i) sequencing coverage (black) and (ii) calculated nucleosome dyads (red, see Methods). The maps have been adjusted so that the total signal for each map is normalised (to unity).

Figure 9

Core histone octamer positioning on genomic DNA sequences at high resolution. The locations and relative abundance of histone octamer binding sites on each genomic DNA, identified as nucleosome dyads, is presented, for all five types of reconstitute, for localised regions of the DNAs. The maps were adjusted so that the total signal for each entire map was normalised (to unity). The numbers indicating nucleosome position on the X axis are on the same scale as shown in Figure 4.

Figure 10

Relationships between histone octamer binding site affinity maps and DNA sequence. Scatter plots of the relative free energy values (ΔG^0) of the binding sites identified from the dyad profiles, measured on each DNA reconstituted with the indicated type of core histone, and the frequency of the AAAA sequence motif within a 147 bp binding site, centred on the dyad of the site, are shown. For each scatter plot the correlation (R) is indicated.

Figure 11

Relationships between histone octamer binding site affinity maps and DNA sequence. The binned data, used to illustrate the relationship between the relative free energy values (ΔG^0) of binding sites and their base composition (Figure 8A), was employed to estimate, from the slopes of linear regression plots, the change in G+C content required to effect a one kcal/mol change in the average free energy (ΔG^0) of a binding site. Analyses for data derived from yeast, Cse4, chicken and frog histone octamer reconstitutes are shown for each of the four DNA types.

Table 1

Edited Bowtie 2 output for the alignment of paired-end reads from the sequencing of nucleosomal DNAs from 5 separate reconstitutions to the Phins reference sequence. Some of the data (blue) was generated using scripts custom written for the purpose.

Table 2a-d

Relationships between histone octamer binding site affinity maps and DNA sequence. The correlation between the relative free energy (ΔG^0) profiles of binding sites and profiles of the frequency of di, tri and tetranucleotide within the binding sites was determined for each DNA type. The data derived from yeast, Cse4, chicken and frog histone octamer reconstitutes was averaged and the standard deviation determined. The data was then sorted to identify the top 30 positively (R) and negatively (-R) correlated sequence motifs. The ranking for G+C (yellow) and AAAA (blue) is identified.

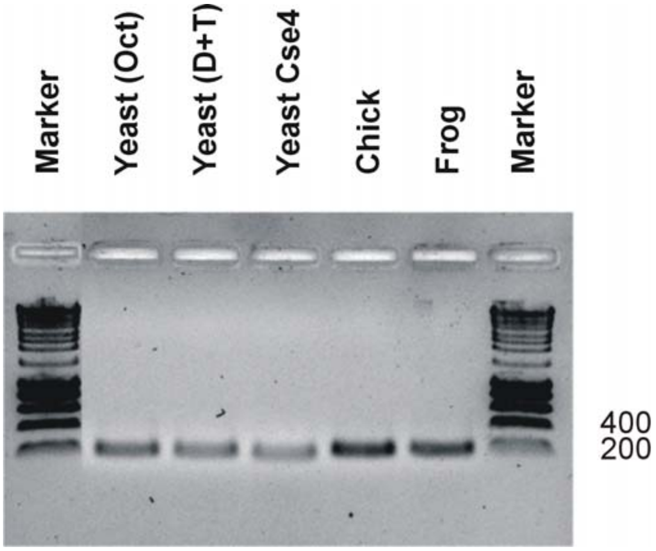

Supplementary Figure 1
(Allan et al, 2013)

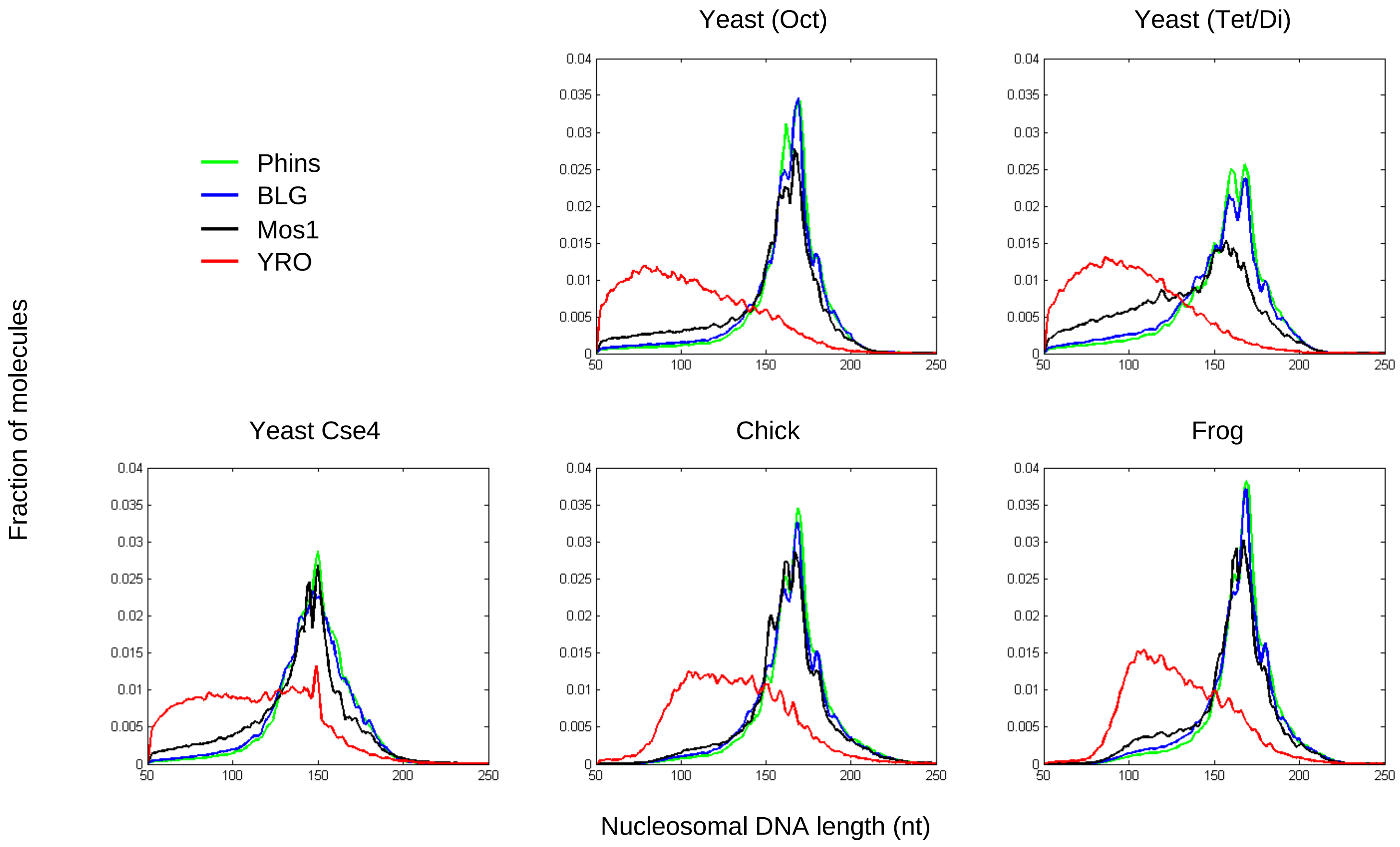

Supplementary Figure 2
(Allan et al, 2013)

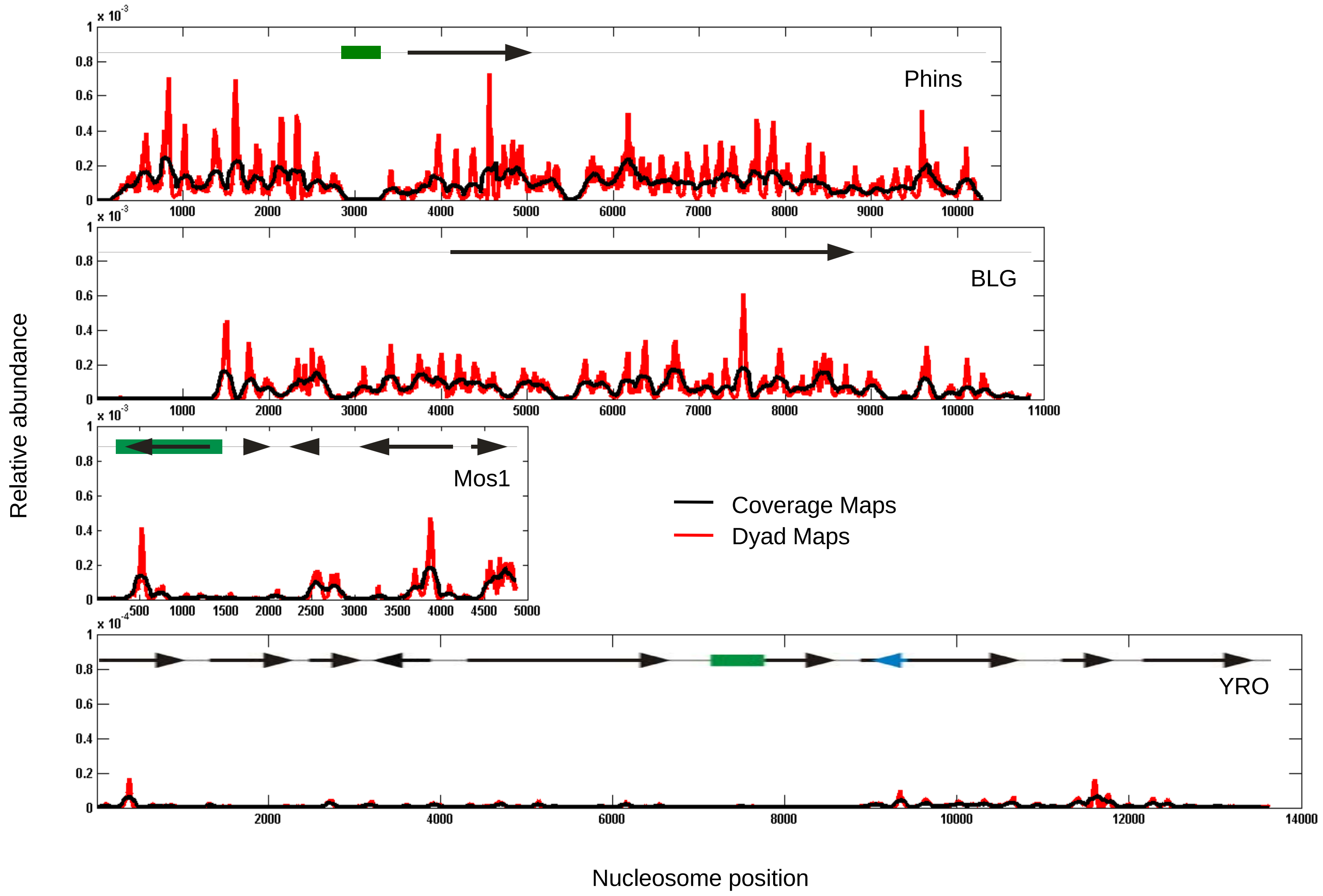

Supplementary Figure 3
(Allan et al, 2013)

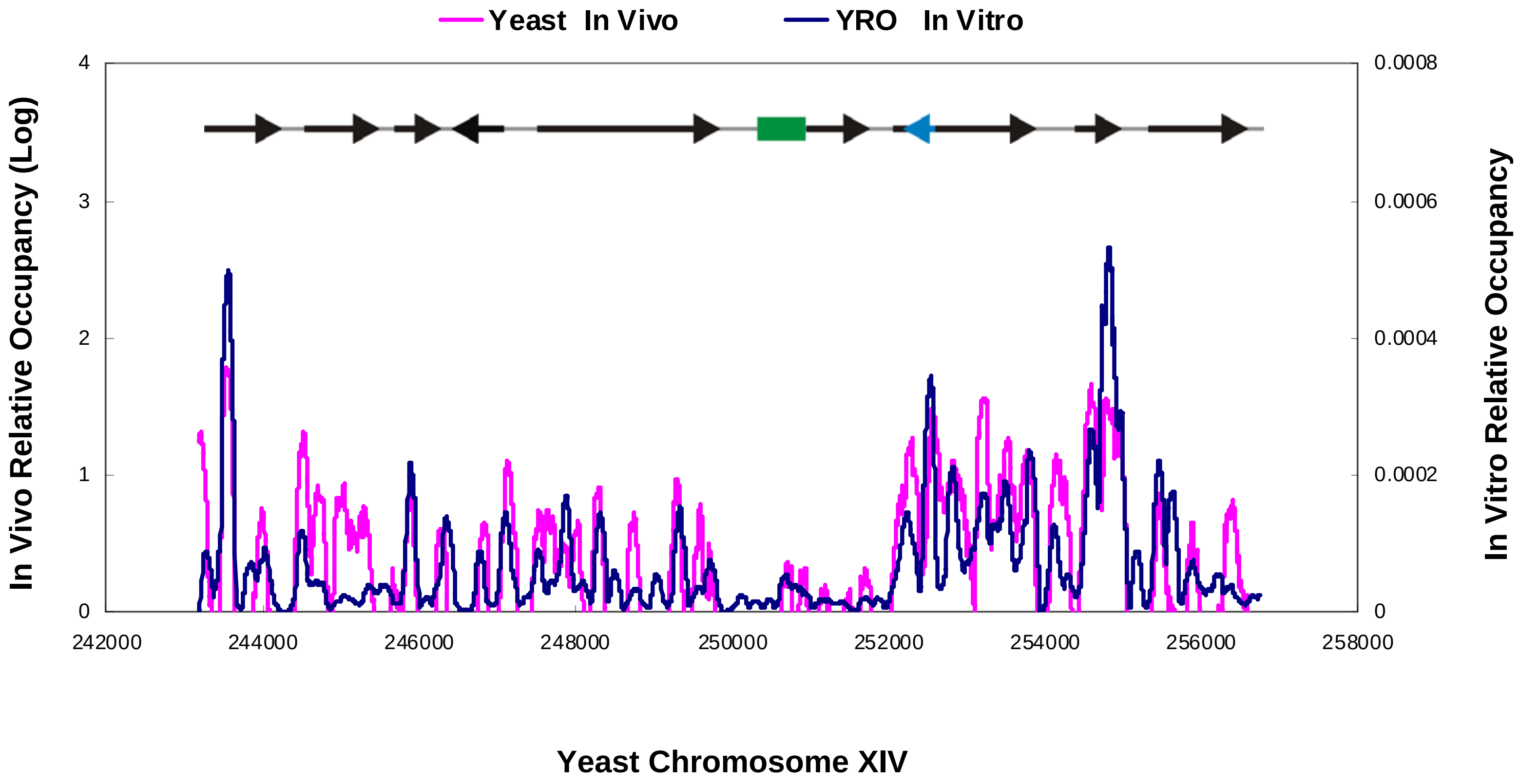

Supplementary Figure 4
(Allan et al, 2013)

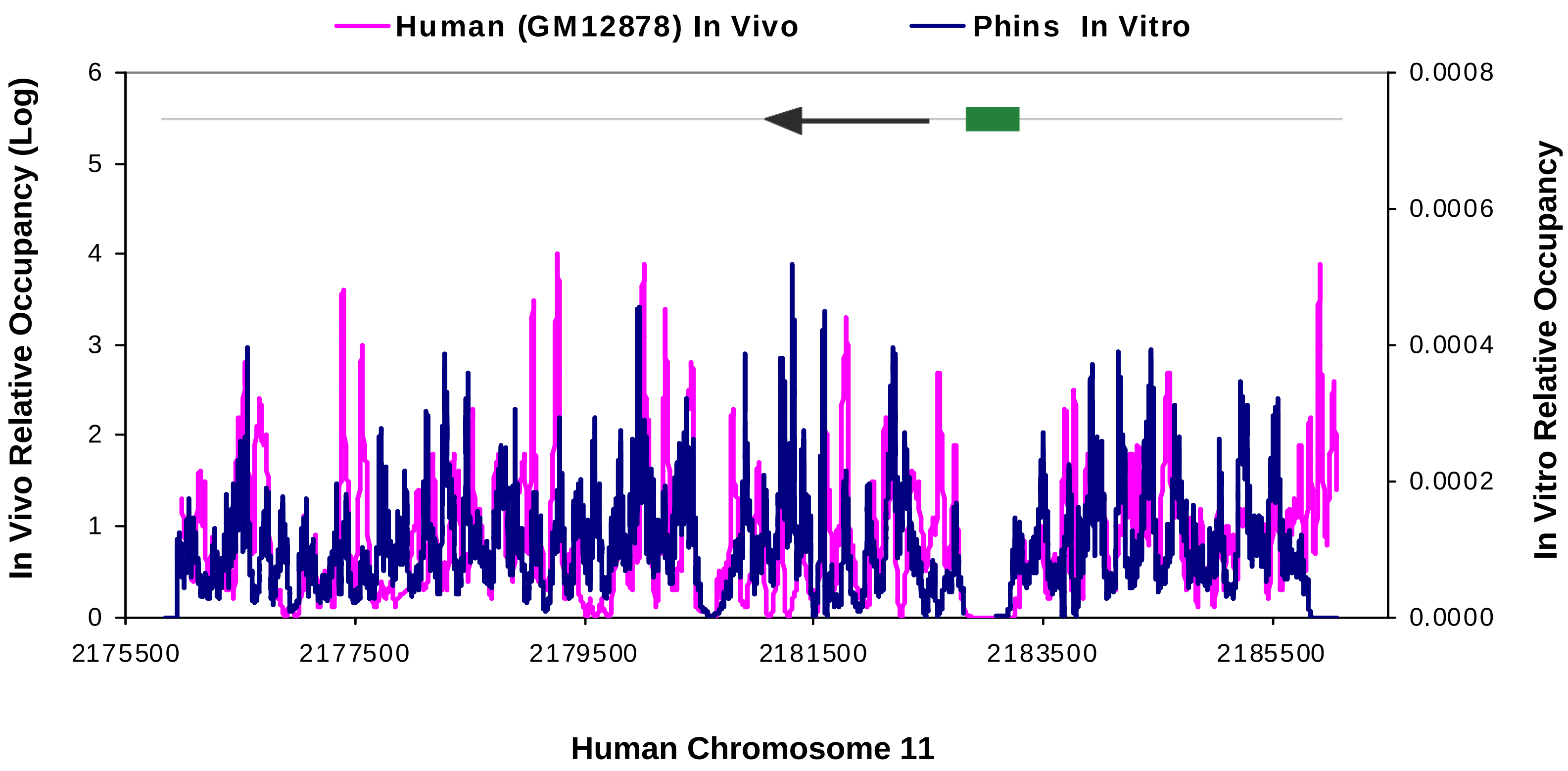

Supplementary Figure 5
(Allan et al, 2013)

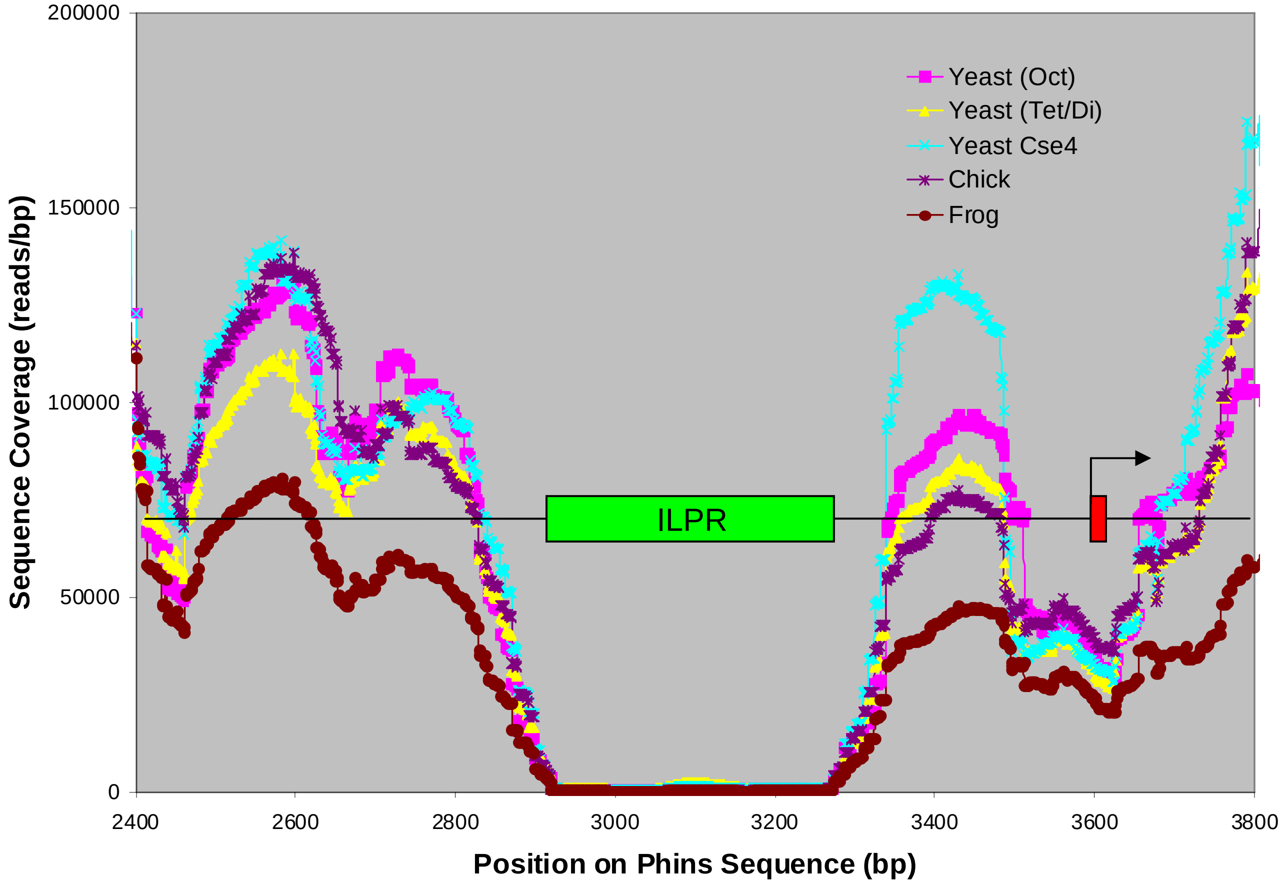

Supplementary Figure 6
(Allan et al, 2013)

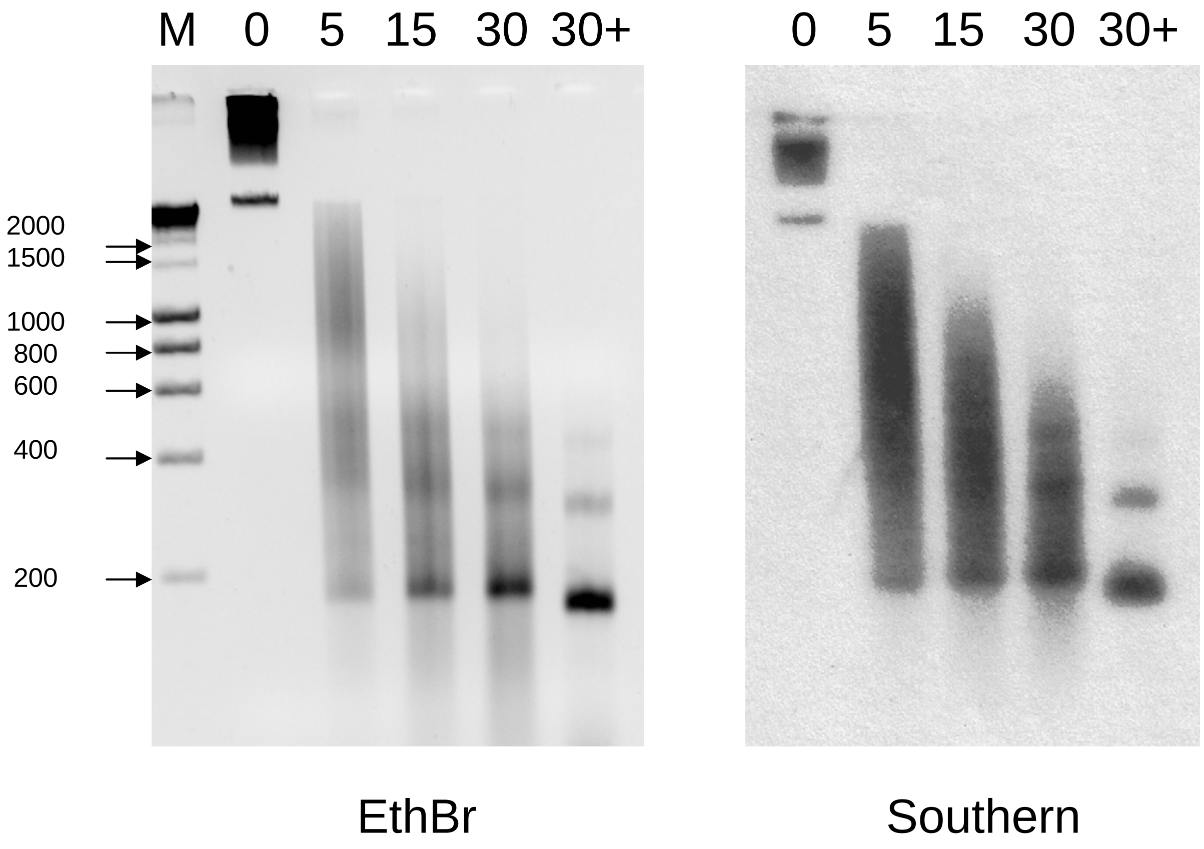

Supplementary Figure 7
(Allan et al., 2013)

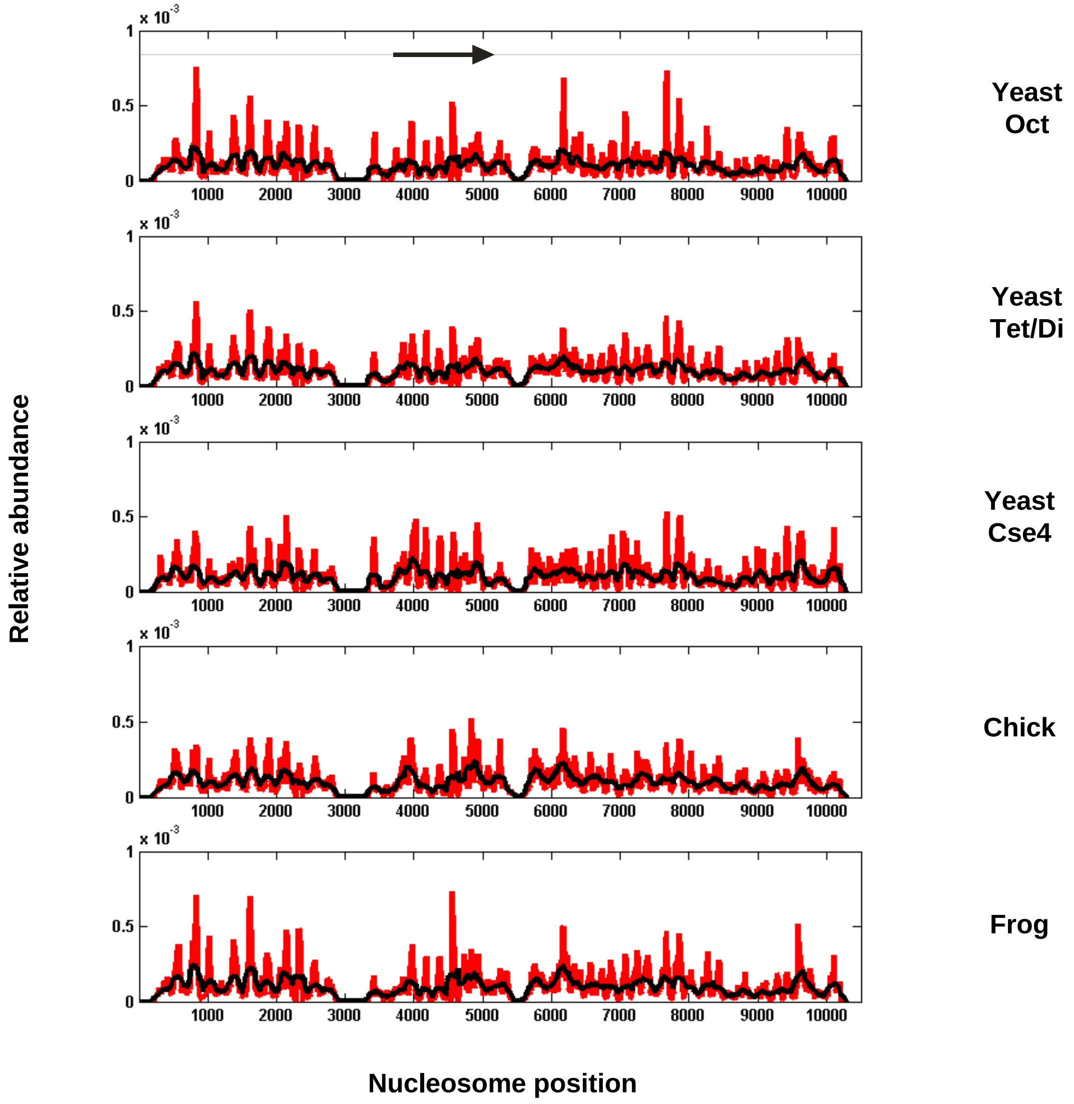

Supplementary Figure 8a
(Allan et al, 2013)

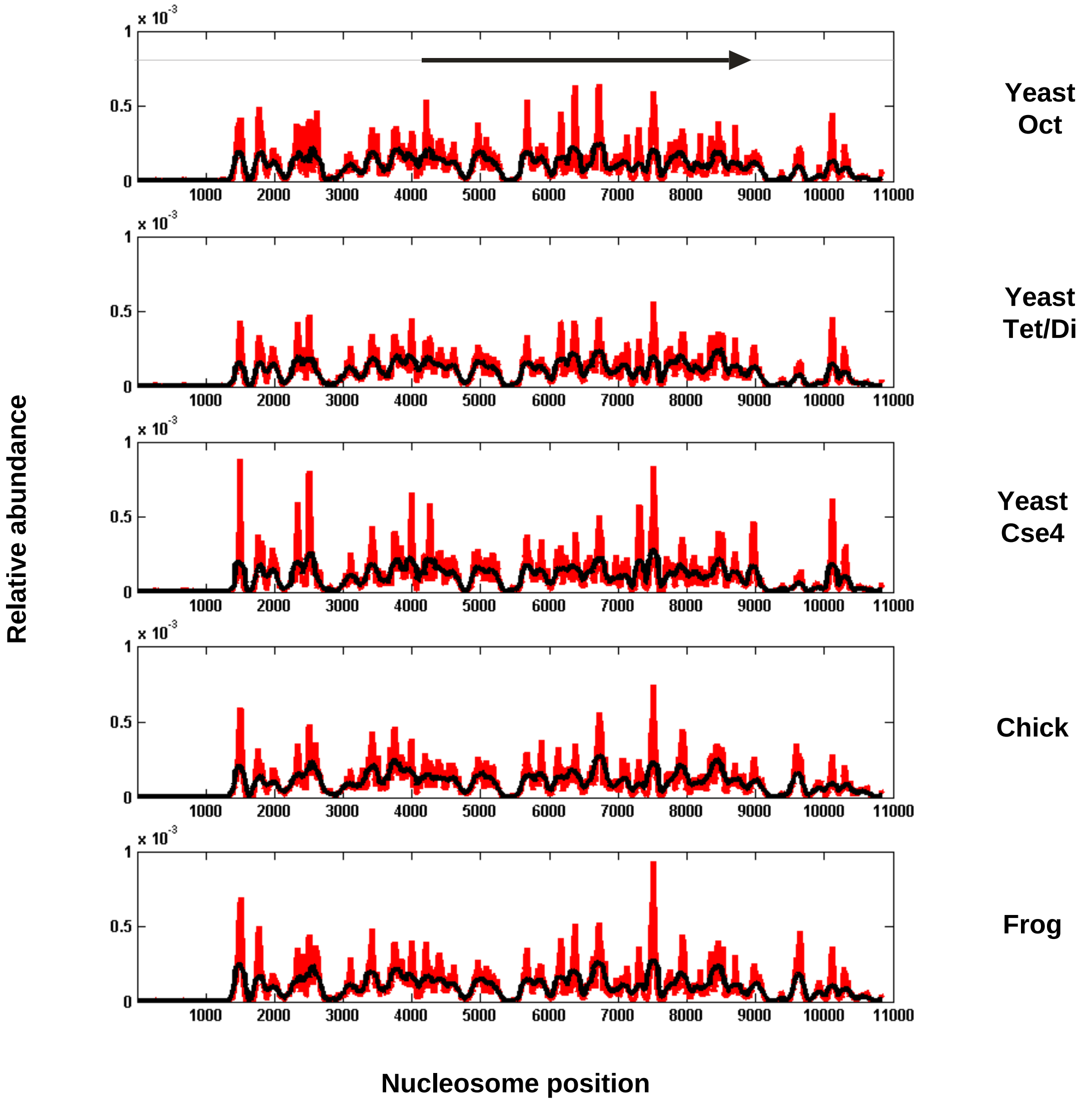

Supplementary Figure 8b
(Allan et al, 2013)

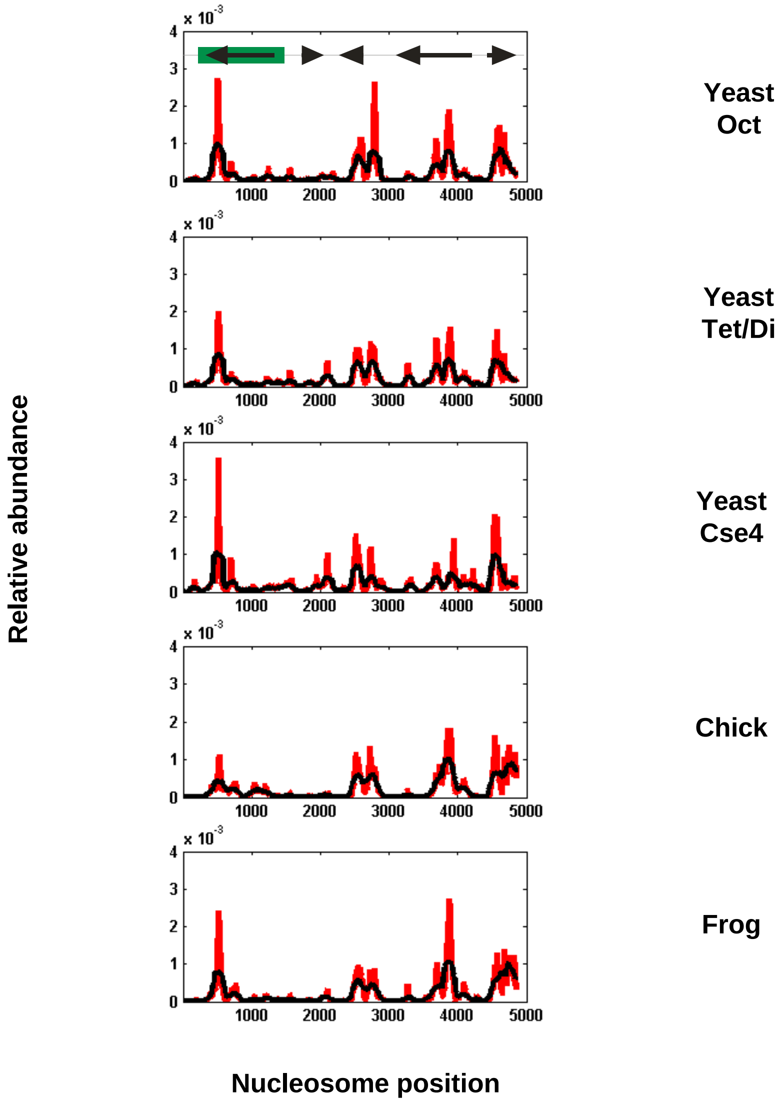

Supplementary Figure 8c
(Allan et al, 2013)

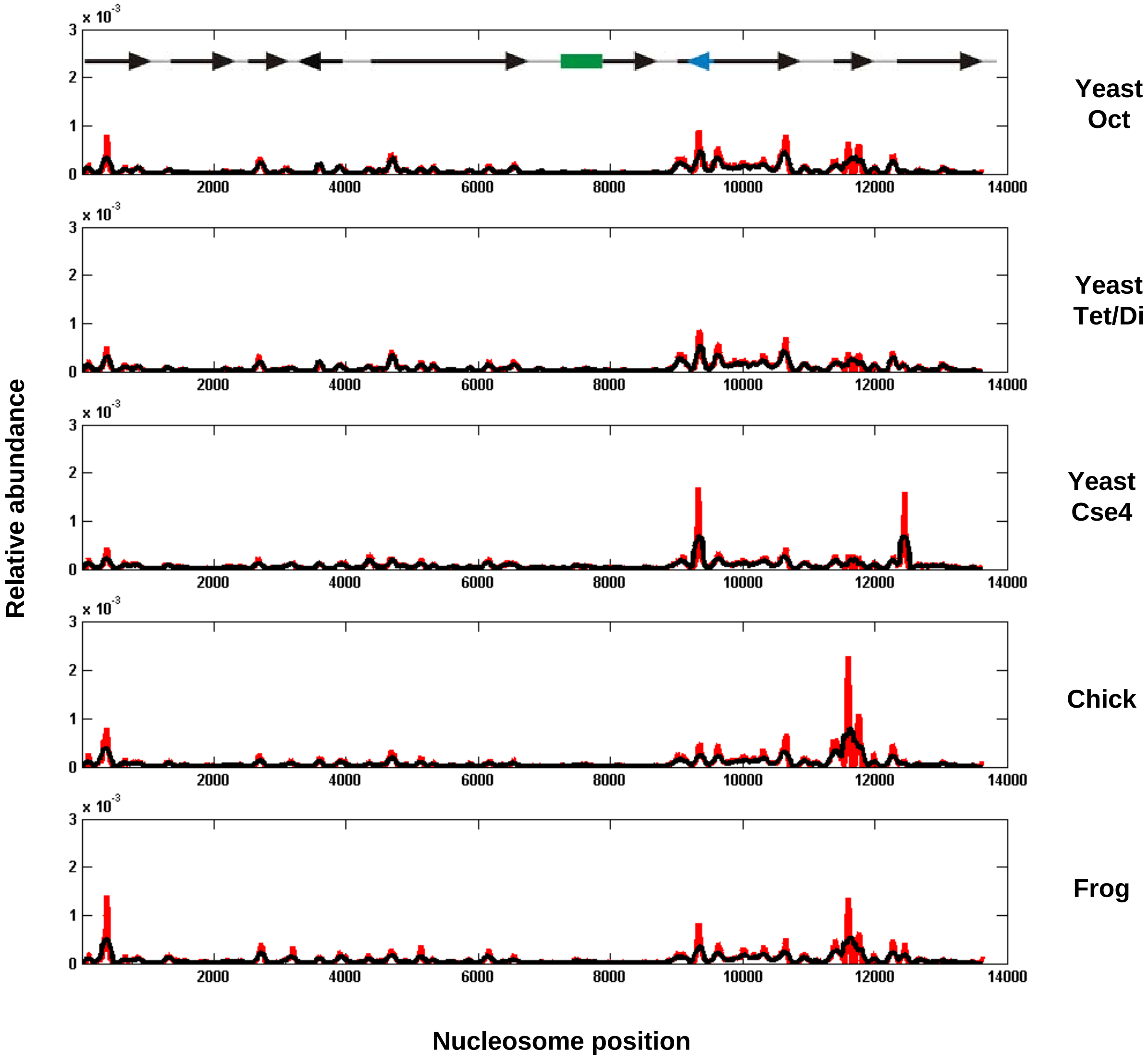

Supplementary Figure 8d
(Allan et al, 2013)

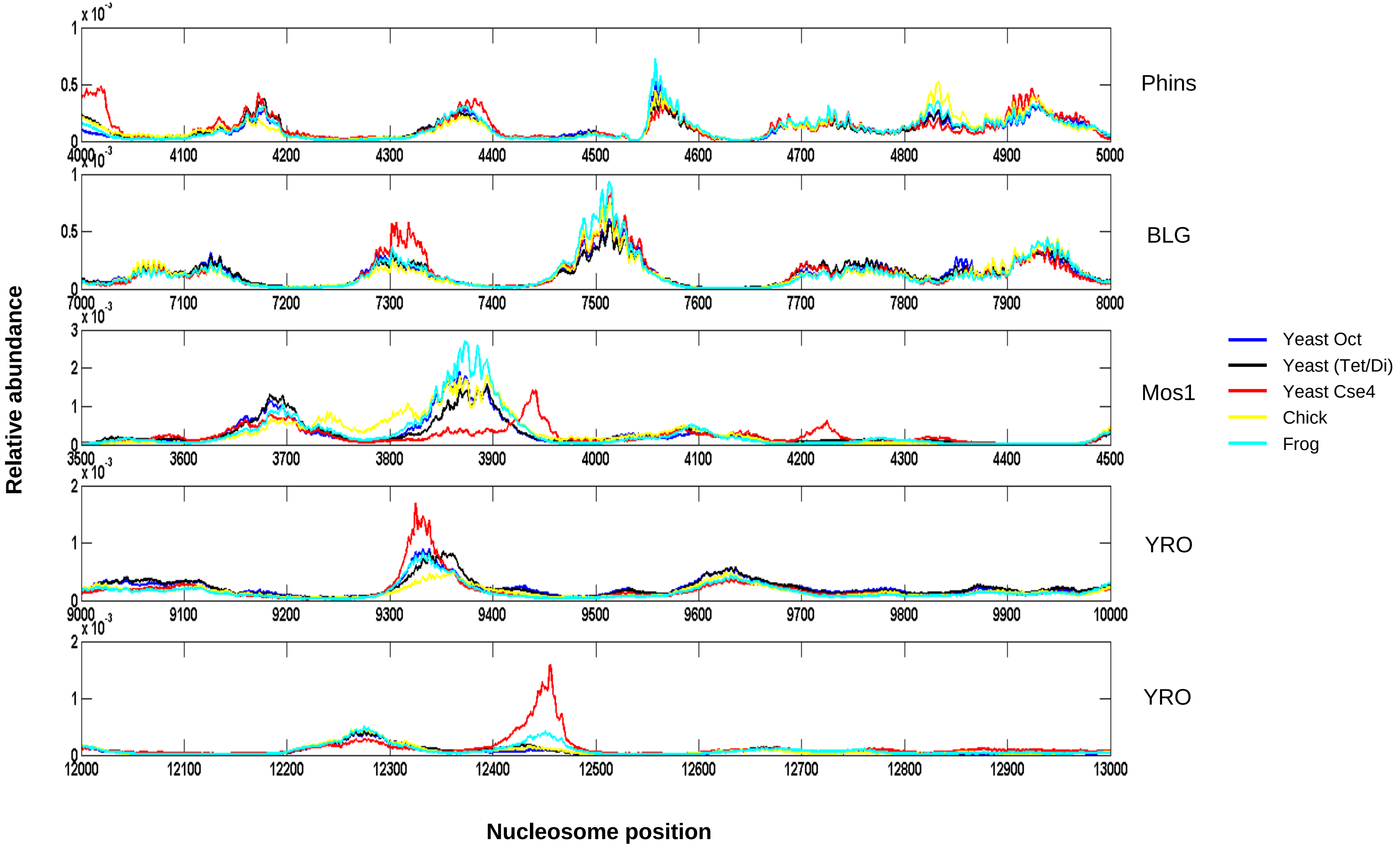

Supplementary Figure 9
(Allan et al, 2013)

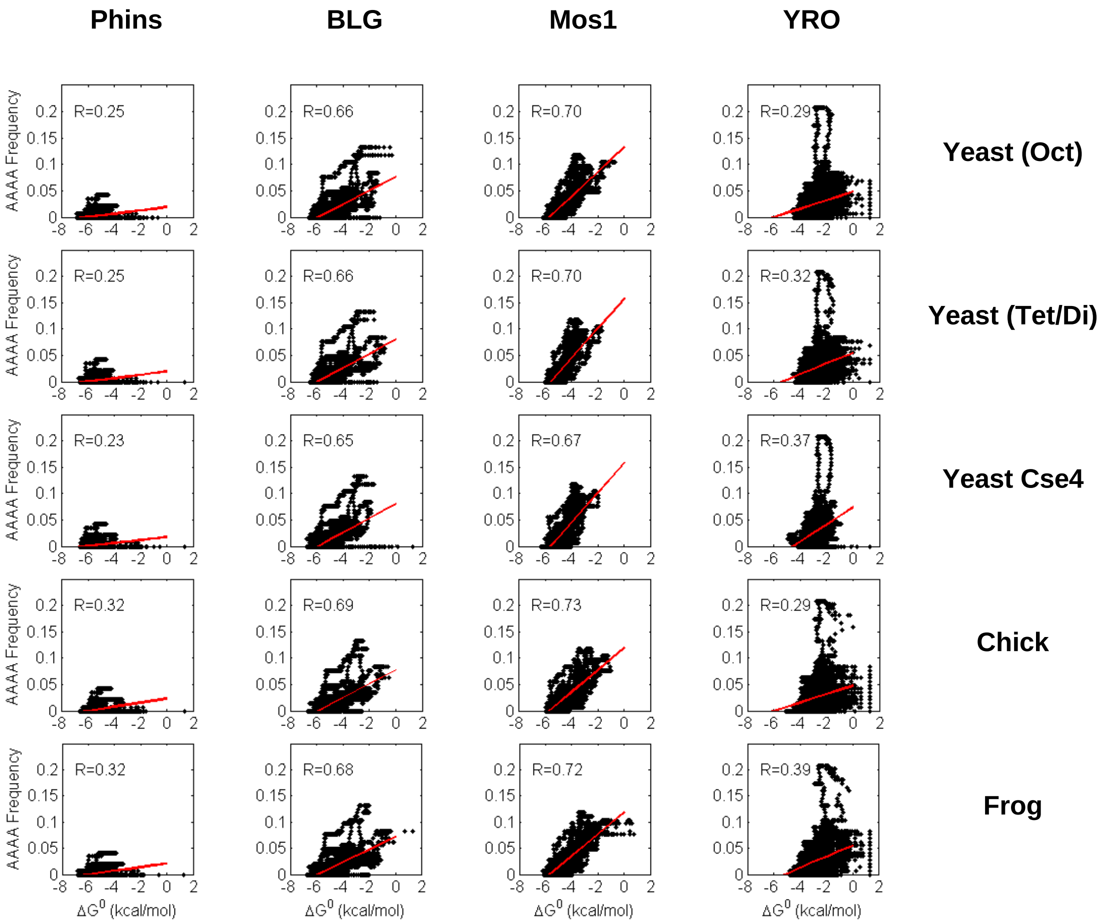

Supplementary Figure 10
(Allan et al, 2013)

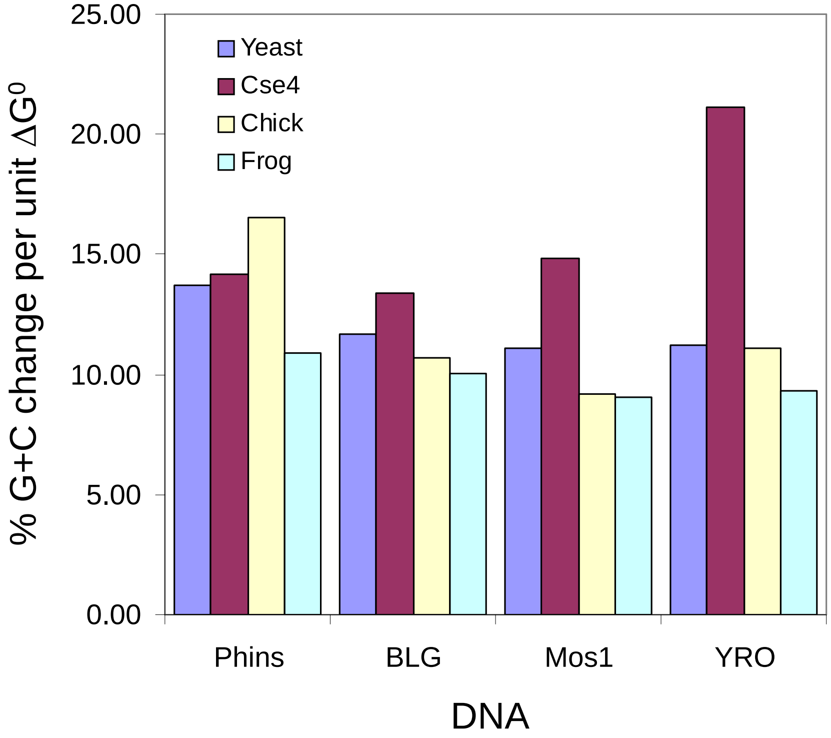

Supplementary Figure 11
(Allan et al, 2013)

Yeast (Oct)
16983429 reads; of these:
16983429 (100.00%) were paired; of these:
10449982 (61.53%) aligned concordantly 0 times
6498452 (38.26%) aligned concordantly exactly 1 time
34995 (0.21%) aligned concordantly >1 times; of these:
34801 (99.45%) aligned to the ILPR; of these:
11.84% aligned to the Forward strand
88.16% aligned to the Reverse strand

Yeast (Tet/Di)
16265713 reads; of these:
16265713 (100.00%) were paired; of these:
9420438 (57.92%) aligned concordantly 0 times
6810296 (41.87%) aligned concordantly exactly 1 time
34979 (0.22%) aligned concordantly >1 times; of these:
34801 (99.45%) aligned to the ILPR; of these:
16.48% aligned to the Forward strand
83.52% aligned to the Reverse strand

Yeast (Cse4)
21932748 reads; of these:
21932748 (100.00%) were paired; of these:
13643497 (62.21%) aligned concordantly 0 times
8244003 (37.59%) aligned concordantly exactly 1 time
45248 (0.21%) aligned concordantly >1 times; of these:
34801 (99.45%) aligned to the ILPR; of these:
9.43% aligned to the Forward strand
90.57% aligned to the Reverse strand

Chick
17541346 reads; of these:
17541346 (100.00%) were paired; of these:
10411663 (59.35%) aligned concordantly 0 times
7096384 (40.46%) aligned concordantly exactly 1 time
33299 (0.19%) aligned concordantly >1 times; of these:
34801 (99.45%) aligned to the ILPR; of these:
4.74% aligned to the Forward strand
95.26% aligned to the Reverse strand

Frog
12367808 reads; of these:
12367808 (100.00%) were paired; of these:
7883393 (63.74%) aligned concordantly 0 times
4464147 (36.09%) aligned concordantly exactly 1 time
20268 (0.16%) aligned concordantly >1 times; of these:
34801 (99.45%) aligned to the ILPR; of these:
6.38% aligned to the Forward strand
93.62% aligned to the Reverse strand

Supplementary Table 1
(Allan et al, 2013)

Phins					
Sequence	Mean	StDev	Sequence	Mean	StDev
GC	-0.355	0.016	A+T	0.346	0.016
C+G	-0.346	0.016	AA	0.343	0.036
CA	-0.283	0.042	AAAT	0.321	0.028
GCCA	-0.272	0.014	AATT	0.320	0.010
GCA	-0.246	0.022	AAT	0.315	0.010
GCC	-0.232	0.014	AAA	0.311	0.046
AGC	-0.208	0.015	ATAG	0.309	0.009
GGCA	-0.206	0.020	GA	0.299	0.019
CAG	-0.205	0.022	GAA	0.287	0.029
CTGC	-0.201	0.016	AGAA	0.284	0.022
CCA	-0.197	0.016	AAAA	0.284	0.038
CAC	-0.187	0.027	ATA	0.275	0.012
GAGC	-0.179	0.011	TA	0.274	0.017
CAGC	-0.170	0.005	GAAA	0.271	0.028
CCAG	-0.165	0.005	TAA	0.246	0.018
GCAC	-0.148	0.017	AATA	0.242	0.014
CAGG	-0.145	0.013	AAGA	0.240	0.015
ACAC	-0.142	0.017	AGA	0.233	0.023
CACC	-0.133	0.027	TAAA	0.228	0.027
ATGC	-0.133	0.021	AT	0.225	0.023
GCCC	-0.128	0.016	AGTC	0.214	0.016
AGCT	-0.126	0.015	ATTC	0.202	0.020
ACCG	-0.126	0.022	AAGT	0.196	0.029
AGCA	-0.119	0.017	TCA	0.192	0.011
CATG	-0.118	0.013	ACTC	0.190	0.017
TGCA	-0.115	0.016	CTAA	0.184	0.018
GCGC	-0.110	0.025	ACT	0.176	0.029
CC	-0.107	0.021	ATAA	0.176	0.011
ACG	-0.106	0.026	ATTA	0.173	0.019
ACGG	-0.103	0.021	AACA	0.152	0.019

Supplementary Table 2a

(Allan et al, 2013)

BLG					
Sequence	Mean	StDev	Sequence	Mean	StDev
C+G	-0.880	0.009	A+T	0.880	0.009
CC	-0.736	0.011	AA	0.866	0.008
GC	-0.718	0.021	AAT	0.843	0.013
CAG	-0.690	0.007	AT	0.822	0.015
GCC	-0.659	0.023	AAA	0.788	0.017
CCA	-0.604	0.006	TA	0.787	0.013
CCC	-0.600	0.014	TAA	0.730	0.009
CAGG	-0.579	0.007	AAAT	0.714	0.017
CCAG	-0.572	0.014	ATA	0.711	0.017
GCCC	-0.551	0.023	AATT	0.682	0.012
CG	-0.525	0.012	AATA	0.677	0.006
AGGG	-0.507	0.009	ATGA	0.648	0.019
AGG	-0.506	0.007	AAAA	0.646	0.020
CCCA	-0.502	0.009	TCAA	0.637	0.019
CAGC	-0.489	0.014	CAA	0.609	0.006
CTGC	-0.472	0.012	GTA	0.605	0.014
CCCC	-0.456	0.012	CAAA	0.595	0.010
CCG	-0.454	0.012	ATTC	0.595	0.018
CAC	-0.453	0.012	GAA	0.585	0.006
AGCC	-0.447	0.013	GATA	0.583	0.021
CACC	-0.439	0.007	AGTA	0.579	0.016
AGC	-0.438	0.013	ATTA	0.578	0.013
GCA	-0.437	0.012	TAAA	0.566	0.012
GGCC	-0.433	0.025	ATTG	0.563	0.013
ACCC	-0.431	0.013	ATCA	0.559	0.017
GGCA	-0.417	0.009	ATAC	0.559	0.024
GCAC	-0.406	0.011	AATG	0.556	0.014
CCCG	-0.403	0.020	TCA	0.538	0.008
AG	-0.399	0.015	ACAA	0.523	0.009
CGC	-0.398	0.013	GAAA	0.523	0.010

Supplementary Table 2b

(Allan et al, 2013)

Mos1					
**Sequence**	**Mean**	**StDev**	**Sequence**	**Mean**	**StDev**
C+G	-0.805	0.077	A+T	0.805	0.077
CC	-0.618	0.062	AA	0.798	0.051
CG	-0.560	0.049	AAA	0.763	0.045
GC	-0.545	0.067	AAAA	0.688	0.024
GCC	-0.523	0.028	AAAT	0.553	0.044
CCGC	-0.498	0.063	TAAA	0.545	0.081
CAC	-0.497	0.049	AAAG	0.518	0.030
CGC	-0.491	0.066	GAAA	0.500	0.028
CCG	-0.480	0.040	TA	0.497	0.080
GCCC	-0.476	0.044	TAA	0.483	0.065
CCC	-0.436	0.043	ATA	0.469	0.075
CCA	-0.432	0.046	AATA	0.459	0.055
CA	-0.427	0.047	AAT	0.451	0.063
CACC	-0.425	0.050	AT	0.446	0.075
CGCC	-0.422	0.034	TTAA	0.395	0.059
GTGA	-0.408	0.040	AGAA	0.377	0.028
GCAC	-0.405	0.054	ATAA	0.376	0.033
GCGA	-0.367	0.031	AAAC	0.369	0.066
CCCA	-0.361	0.054	ATTA	0.369	0.043
ATTC	-0.351	0.019	ATAT	0.343	0.062
CGA	-0.349	0.009	AAGT	0.330	0.059
ACC	-0.347	0.041	TATA	0.322	0.053
GA	-0.341	0.031	CATA	0.311	0.061
CTC	-0.340	0.019	CAAA	0.296	0.023
ACTC	-0.334	0.029	AAG	0.290	0.011
GCCA	-0.332	0.044	ACAT	0.272	0.055
ACCC	-0.317	0.051	TACA	0.260	0.042
CCAC	-0.313	0.058	AATT	0.259	0.027
CCCG	-0.312	0.050	ATAC	0.219	0.032
CAG	-0.305	0.046	ATAG	0.206	0.072

Supplementary Table 2c

(Allan et al, 2013)

YRO					
**Sequence**	**Mean**	**StDev**	**Sequence**	**Mean**	**StDev**
C+G	-0.593	0.073	A+T	0.593	0.073
CA	-0.415	0.021	TA	0.490	0.105
GC	-0.364	0.018	ATA	0.479	0.093
CG	-0.357	0.031	AT	0.455	0.071
CAGC	-0.350	0.005	TATA	0.449	0.091
GAC	-0.340	0.023	AAT	0.409	0.040
ACC	-0.339	0.026	ATAT	0.387	0.085
CCA	-0.327	0.051	AA	0.373	0.063
CC	-0.321	0.069	AAA	0.367	0.052
CAG	-0.313	0.025	TAA	0.366	0.068
ACCG	-0.308	0.026	TAAA	0.359	0.057
CCAA	-0.301	0.017	AATA	0.340	0.062
AACC	-0.286	0.022	AAAT	0.330	0.042
AGC	-0.281	0.020	ATTA	0.320	0.054
CGAC	-0.277	0.008	AAAA	0.303	0.043
CAA	-0.276	0.033	ATAA	0.283	0.036
GCA	-0.275	0.008	TACA	0.274	0.053
CCG	-0.275	0.030	ACTA	0.261	0.064
CGA	-0.275	0.021	ATAG	0.261	0.065
CAAG	-0.271	0.021	AATG	0.240	0.014
AGCA	-0.259	0.010	GAAA	0.232	0.042
GGCA	-0.254	0.014	ATAC	0.212	0.041
CCAG	-0.240	0.041	TTAA	0.205	0.053
CTGC	-0.237	0.008	AGTA	0.195	0.033
TCGA	-0.234	0.026	GTA	0.190	0.058
CAAC	-0.233	0.008	CTA	0.173	0.069
GCC	-0.231	0.044	GTAA	0.165	0.039
AGCG	-0.225	0.019	GGAA	0.158	0.024
ACCA	-0.217	0.011	CCTC	0.148	0.073
GGAC	-0.216	0.034	CATA	0.143	0.029

Supplementary Table 2d

(Allan et al, 2013)
